# QTLs and candidate genes for desiccation and abscisic acid content in maize kernels

**DOI:** 10.1186/1471-2229-10-2

**Published:** 2010-01-04

**Authors:** Valérie Capelle, Carine Remoué, Laurence Moreau, Agnès Reyss, Aline Mahé, Agnès Massonneau, Matthieu Falque, Alain Charcosset, Claudine Thévenot, Peter Rogowsky, Sylvie Coursol, Jean-Louis Prioul

**Affiliations:** 1 Univ Paris-Sud, Institut de Biotechnologie des Plantes, Bât 630, F-91405 Orsay, France; 2 CNRS, UMR 8618, F-91405 Orsay, France; 3 CNRS, UMR 0320/UMR 8120 Génétique Végétale, F-91190 Gif-sur-Yvette, France; 4 INRA, UMR 0320/UMR 8120 Génétique Végétale, F-91190 Gif-sur-Yvette, France; 5 INRA, Reproduction et Développement des Plantes, UMR 879 INRA-CNRS-ENSL-UCBL, IFR128 Biosciences Lyon-Gerland, F-69364 Lyon Cedex 07, France; 6 52, Av de la Marjolaine, 34110 Frontigan, France

## Abstract

**Background:**

Kernel moisture at harvest is an important trait since a low value is required to prevent unexpected early germination and ensure seed preservation. It is also well known that early germination occurs in viviparous mutants, which are impaired in abscisic acid (ABA) biosynthesis. To provide some insight into the genetic determinism of kernel desiccation in maize, quantitative trait loci (QTLs) were detected for traits related to kernel moisture and ABA content in both embryo and endosperm during kernel desiccation. In parallel, the expression and mapping of genes involved in kernel desiccation and ABA biosynthesis, were examined to detect candidate genes.

**Results:**

The use of an intermated recombinant inbred line population allowed for precise QTL mapping. For 29 traits examined in an unreplicated time course trial of days after pollination, a total of 78 QTLs were detected, 43 being related to kernel desiccation, 15 to kernel weight and 20 to ABA content. Multi QTL models explained 35 to 50% of the phenotypic variation for traits related to water status, indicating a large genetic control amenable to breeding. Ten of the 20 loci controlling ABA content colocated with previously detected QTLs controlling water status and ABA content in water stressed leaves. Mapping of candidate genes associated with kernel desiccation and ABA biosynthesis revealed several colocations between genes with putative functions and QTLs. Parallel investigation via RT-PCR experiments showed that the expression patterns of the ABA-responsive *Rab17 *and *Rab28 *genes as well as the late embryogenesis abundant *Emb5 *and aquaporin genes were related to desiccation rate and parental allele effect. Database searches led to the identification and mapping of two *zeaxanthin epoxidase *(*ZEP*) and five novel *9-cis-epoxycarotenoid dioxygenase *(*NCED*) related genes, both gene families being involved in ABA biosynthesis. The expression of these genes appeared independent in the embryo and endosperm and not correlated with ABA content in either tissue.

**Conclusions:**

A high resolution QTL map for kernel desiccation and ABA content in embryo and endosperm showed several precise colocations between desiccation and ABA traits. Five new members of the maize *NCED *gene family and another maize *ZEP *gene were identified and mapped. Among all the identified candidates, aquaporins and members of the *Responsive to ABA *gene family appeared better candidates than *NCEDs *and *ZEPs*.

## Background

Maize (*Zea mays*) kernel moisture at harvest is an important trait in temperate regions because costly additional drying is needed to reach 15% water content, which is the level compatible with good seed preservation during storage. Although yield is correlated to maximum water content occurring approximately 40-60 days after pollination (DAP) [1] and to a lesser extent to kernel moisture at maturity [2], the yield/moisture ratio at maturity is variable enough to allow selection for both higher yield and lower moisture at harvest [3,4]. For example, recurrent selection has been successfully applied for reduction of kernel moisture by the introduction of tropical germplasm into temperate-adapted germplasm [5]. Indirect inbred selection criteria to reduce gain moisture, based on husk senescence have been proposed [6]. The biochemical, biophysical and molecular phenomena controlling kernel moisture at harvest intervene mainly during the maturation phase, which corresponds to the last stage of seed development after the early and the grain filling phases. Physiological and genetic analyses of the maturation phase reveal a process, which both prevents early embryo germination and favours the synthesis of specialized proteins related to the acquisition of desiccation tolerance, enhancing embryo viability under strong dehydration. Accordingly, in many seed species including maize, embryos separated from endosperm at the early developmental phase can grow and germinate when placed in tissue culture, but their germination ability decreases as maturation proceeds [7].

Despite considerable progress in recent years in knowledge of maturation, the number of genes thought to be involved in regulation of kernel moisture remains extremely limited. The late embryogenesis abundant (LEA) proteins including the dehydrin family, are specifically produced during the maturation phase [8]. Although they have been assumed for a long time to protect cellular and molecular structures from the damaging effect of desiccation [8], only recent results shed some light on their precise action. Cytoplasmic LEA proteins prevent protein aggregation due to water loss *in vitro *[9] and mitochondria LEA proteins protect two matrix enzymes, fumarase and rhodanese [10]. Other proteins likely to be involved in kernel desiccation are the water channel aquaporins, which are present in nearly all organs. An extensive study of maize aquaporins described 31 full length cDNAs distributed into four groups comprising 13 plasma membrane (PIP) and 11 tonoplast (TIP) intrinsic proteins [11]. Among them, *ZmPIP1;1*, *ZmPIP1;2*, *ZmPIP1;3*, *ZmPIP2;1*, *ZmPIP2;2*, *ZmPIP2;3*, *TIP1;1 *and *TIP2;1 *were reported to be expressed in reproductive tissues. In rice (*Oryza sativa*), *OsTIP1 *and *OsTIP3 *are expressed in mature seeds in the embryo and the aleurone layer, respectively. Because members of the PIP2 and TIP1 families have much higher water transport capacities than those of the PIP1 family [12], aquaporins of the first two families may be of higher significance for desiccation.

The phytohormone abscisic acid (ABA) appears to play a central role in both the establishment of embryo dormancy and the synthesis of LEA proteins, as demonstrated by mutants impaired in ABA synthesis or sensitivity. ABA deficient maize mutants are viviparous, i.e. embryos germinate precociously on the ear [13,14], their vivipary being prevented by ABA addition. ABA synthesis mutants of Arabidopsis (*Arabidopsis thaliana*) and tomato (*Solanum lycopersicum*) have also impaired seed maturation and dormancy but are not viviparous [15]. Interestingly, maize plants with white endosperm (*yellow *mutants) have higher moisture than those with yellow endosperm [16]. This is due to the fact that the *y1 *mutation causes a defect in phytoene synthase (PSY), an enzyme involved in both carotenoid and ABA synthesis [17], highlighting the likely role of ABA in regulating kernel moisture. Furthermore, the expression of many *LEA *genes and more generally members of the *Responsive to ABA *(*Rab*) gene family, is induced by exogenous ABA [18].

The ABA biosynthetic and catabolic pathways are now well understood since almost all the biosynthetic genes have been identified through the isolation of auxotrophic mutants [19]. The enzymes downstream of the xanthophyll cycle are specific to ABA biosynthesis. The cloning and characterization of maize *Viviparous14 *(*Vp14*), which encodes 9-*cis*-epoxycarotenoid dioxygenase 1 (NCED1) catalyzing the cleavage of the C40 neoxanthin chain into the C15 ABA skeleton xanthoxin [20], led to the identification of NCED as a rate controlling enzyme. Indeed, maize *nced1 *mutants have a strongly reduced kernel ABA content [21] and in Arabidopsis, *NCED1 *overexpression confers a significant increase in ABA accumulation in the plant [22]. In Arabidopsis, nine *NCED*-related sequences have been identified and phylogenetic analysis has indicated that five of these clustered with functionally characterized NCED proteins from other species [23]. Aside from this main regulatory step in the ABA biosynthesis pathway, other metabolic steps of ABA metabolism also contribute to determining ABA level. One is the conversion of zeaxanthin into violaxanthin catalyzed by zeaxanthin epoxidase (ZEP), which is encoded by a single-copy gene in Arabidopsis and tobacco (*Nicotiana plumbaginifolia*) and whose overexpression causes an enhanced accumulation of ABA in seeds [24]. The cloning and characterization of the maize *Viviparous10/Viviparous13 *(*Vp10*/*Vp13*) and *Viviparous15 *(*Vp15*) genes further demonstrated that ABA biosynthesis is also dependent on a molybdenum cofactor involved in the abscisic aldehyde oxidase reaction, the last step of ABA biosynthesis [25,26]. In addition, the recent discovery that *Viviparous8 *(*Vp8*) encodes a putative peptidase, together with the predominant effect of *vp8 *mutant on ABA synthesis and turnover in maize embryos, indicate that ABA level is also controlled indirectly through regulation of seed-specific factors [27].

The mutant approach is powerful in identifying the mandatory steps (genes) in a pathway, but it does not provide any insight into the relative impact of each step on the quantitative value of the final product (trait) of the pathway. The genetic variability of quantitative traits is controlled by one or generally several loci named quantitative trait loci (QTLs) which may be mapped using appropriate segregating populations. In addition, the relative contribution of each locus to the trait genetic variation and the allelic effect at each locus may be estimated. More than 20 years ago, Robertson proposed a very fruitful hypothesis bridging mutation and QTL approaches by simply saying that the "qualitative and quantitative traits may be the result of different types of variation of genic DNA at the loci involved"; in other words minor allelic effects produce quantitative variations, while major variations (null alleles) produce qualitative variations (mutations) [28]. This opened the way for research aiming at the identification of the genes underlying QTLs. The considerable international efforts in mapping known function genes in maize now provide rather precise genetic maps that can be used to identify candidate genes from their map common location with detected QTLs [29]. This comparison is easier when dealing with physiological and biochemical traits since the number of possible candidate genes may be restricted to those encoding enzymes or cofactors acting in relevant pathways [30]. However, one limitation is the confidence interval of the QTL position which may reach more than 10 centimorgans (cM) in classical recombinant inbred lines (RILs) as illustrated in one of the few reports on QTL for kernel moisture and drying rate [31]. A way to reduce this interval is to increase the number of recombination events by the inclusion of four generations of random intermating after the second generation and before the single seed descent, thus providing intermated recombinant inbred lines in which the QTL confidence interval is substantially reduced by a factor of two to three [32]. The candidate gene selection is thus facilitated. A useful criterion to validate the identified candidate genes is to examine the corresponding gene expression during the process under investigation [33]. Differences in the transcription level related to the trait variation may support the role of the functional difference of the parental alleles.

Much remains to be learned about the genes explaining the variability in the desiccation rate and the genetic relationship between this process and ABA content. Here, we describe a QTL-candidate gene analysis of the desiccation process in maize using an intermated recombinant inbred line population (LHRF_F_3:4_) derived from the cross between the maize inbred lines F2 and F252 differing in desiccation rate. First, QTLs were searched for traits related to kernel moisture and ABA content in the endosperm and the embryo during kernel desiccation. Second, an extensive data mining of the genes mapped in the confidence interval of the QTLs was performed in order to short list candidate genes with annotations related to desiccation rate and/or ABA content. In addition to these *in silico *studies, six members of the *NCED *gene family and two members of the *ZEP *gene family were identified and mapped by PCR amplification and sequencing. Third, expression profiles of the candidate genes during desiccation were examined by RT-PCR experiments for correlations with kernel desiccation rates or changes in ABA content.

## Results

### Genetic variability in desiccation rate and ABA content

**Figure 1:**
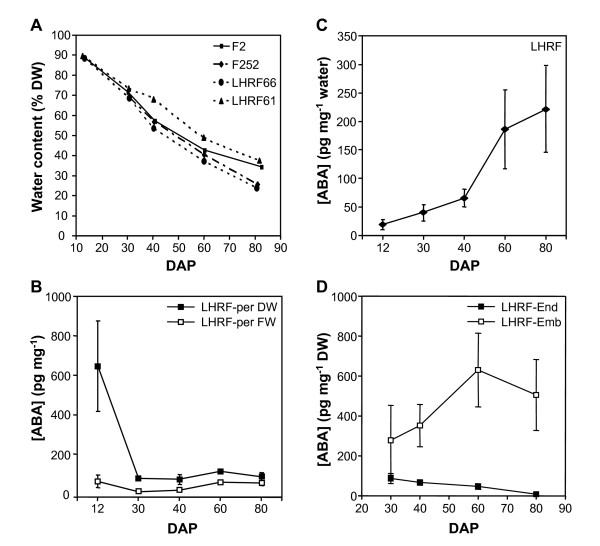
**Time course of mean water status and ABA content in kernel in parents and inbreds**. The LHRF_F_3:4_segregating population derived from the cross between the F2 and the F252 parental inbred lines differing in desiccation rate (four intermated cycle were performed after the second generation and before single descent). A, water content expressed as a percentage of dry weight (% DW) in the two parental inbred lines and the best (LHRF61) and worst (LHRF66) LHRF_ F_3:4_families. B, ABA concentration per kernel dry matter weight (DW) or per kernel fresh matter weight (FW) in the LHRF_ F_3:4_population (LHRF). C, ABA concentration per kernel water in the LHRF_ F_3:4_population (LHRF). D, ABA concentration per dry matter weight (DW) in endosperm (End) and embryo (Emb) of the LHRF_ F_3:4_population (LHRF). Data from LHRF are means ± SD; *n *= 153.

Kernel water content relative to dry weight (%DW) was evaluated in the two parental inbred lines and the segregating LHRF_F_3:4 _population. This trait continuously declined from 12 DAP when the kernel was still in the filling stage and long before the onset of the maturation stage at 30 DAP (Fig. 1A, Table 1). However, kernel water content (g/kernel) reached a maximum between 30 to 40 DAP (data not shown). This maximum corresponded to the end of the intensive starch accumulation and indicated the beginning of the desiccation-maturation process. Thus, further data presentation was limited to the 30-80 DAP period. The two parental lines had different desiccation rates, especially after 40 DAP, F252 line having approximately 9% less moisture ((FW - DW)/FW*100) than F2 line at harvest (Fig. 1A, Table 1).

**Table 1:** Characteristics of the parental lines and their offspring for desiccation rate and ABA content

Trait	LHRF_F_3:4_ mean ± SD	LHRF_F_3:4_ min-max	F2 mean ± SD	F252 mean ± SD
FW30	188 ± 33	123-395	183 ± 14	180 ± 20
FW40	247 ± 36	156-351	260 ± 17	249 ± 19
FW60	316 ± 44	217-445	332 ± 36	319 ± 13
FW80	306 ± 50	189-450	314 ± 38	295 ± 33
DW30	55 ± 9	34-87	52 ± 7	55 ± 4
DW40	103 ± 14	67-146	111 ± 7	108 ± 9
DW60	183 ± 24	122-259	190 ± 20	190 ± 9
DW80	208 ± 30	138-288	206 ± 25	220 ± 18
%DW30	30 ± 4	17-39	29 ± 2	31 ± 1
%DW40	42 ± 3	32-49	43 ± 2	43 ± 1
%DW60	58 ± 3	46-66	57 ± 2	60 ± 1
%DW80	68 ± 4	58-78	66 ± 2	74 ± 3
Water30	133 ± 28	87-308	131 ± 8	124 ± 16
Water40	143 ± 25	85-222	149 ± 13	142 ± 10
Water60	132 ± 23	82-193	142 ± 19	128 ± 5
Water80	98 ± 24	43-165	108 ± 16	75 ± 16
ABAend30	87 ± 25	41-200	93 ± 8	114 ± 19
ABAend40	67 ± 14	38-121	72 ± 33	76 ± 5
ABAend60	47 ± 14	17-92	75 ± 27	85 ± 15
ABAend80	8 ± 4	2-21	79 ± 28	31
ABAemb30	278 ± 175	42-1004	273	212 ± 3
ABAemb40	352 ± 106	158-714	489 ± 56	408 ± 44
ABAemb60	630 ± 184	290-1356	619 ± 152	380 ± 0.2
ABAemb80	505 ± 178	115-1091	544 ± 105	350 ± 60
Slope	-0.083 ± 0.006	-0.100-0.070	-0.071	-0.085
Rate30_80	0.082 ± 0.006	0.060-0.096	0.074	0.086
Rate30_40	0.124 ± 0.045	0.020-0.240	0.142	0.119
Rate40_60	0.082 ± 0.020	0.010-0.130	0.072	0.084
Rate60_80	0.050 ± 0.018	0.010-0.110	0.042	0.073

The LHRF_F_3:4_segregating population derived from the cross between the F2 and the F252 parental inbred lines differing in desiccation rate. FW: kernel fresh matter weight (mg/kernel); DW: kernel dry matter weight (mg/kernel); %DW = DW/FW× 100; Water: kernel water content (mg/kernel); ABAend: ABA in endosperm (pg/DW); ABAemb: ABA in embryo (pg/DW); Slope: regression line slope of (Water/FW × 100) as a function of thermal time; Rate = (Water/FW × 100)/(thermal time interval).

Mean ABA concentration in the LHRF_F_3:4 _population, when expressed on DW basis, declined drastically from 12 to 30 DAP and then, increased slightly up to 60 DAP (Fig. 1B). Similar kinetics albeit with a much lower amplitude between 12 and 30 DAP was observed when ABA was expressed on FW basis, whereas the ABA content per mg of water increased continuously and markedly after 40 DAP (Fig. 1C). Because ABA is water soluble, the later mode of expression was likely the most physiologically relevant, but also the most difficult to obtain when working with lyophilized powder as in the present experiments. The interpretation of global changes at the kernel level was further complicated by large differences between the different kernel parts, ABA being 5 to 60 times more concentrated in the embryo than in the endosperm (Table 1). In addition, the kinetics in the two tissues was also clearly different. In the embryo, the bell-shaped ABA concentration curve peaked at 60 DAP and remained high at 80 DAP. In contrast, ABA concentration continuously declined in the endosperm (Fig. 1D). Large genotypic variability among the 153 LHRF_F_3:4 _lines was noted in the general trend as shown by the large standard deviations. Principal component analysis and Pearson coefficient tables with all the measured variables showed that FW was highly correlated to DW or kernel water content (*r *> 0.69 to 0.94) at a given DAP stage, but not between stages (Additional file 1). By contrast, regardless of the considered stage, the correlation between endosperm ABA content and embryo ABA content was not significant or very low (*r *= 0.23). This was also true across DAP stages (Additional file 1). Some low but significant correlations were noted between some water-related variables and ABA content in the endosperm or the embryo at a same DAP stage (e.g. ABAemb80 and FW80, Additional file 1).

The trait variability was higher in the inbred lines than in parental lines, which is usual with a complex trait, the best line having lower moisture than F252 line and the worst line having higher moisture than F2 line (Fig. 1A for water content; Table 1 for others traits), illustrating the so-called transgression effect. The existence of positive and negative allelic effects for each trait is in favour of a genotypic origin of the transgressions observed at the phenotypic level (Table 2).

**Table 2:** QTLs detected for kernel desiccation and ABA content at 30, 40, 60 and 80 DAP

Number	QTL name	Chr.bin	Position	ProxMarker	DistMarker	Confidence interv.+/- LOD = 1	LOD value	R^2^	Additive value/F252
1	FW_30_1	4.03	88	nc004	umc2039	76-104	2.54*	7.5	-13.203

2	FW_40_1	5.05	396	umc1822	umc54	372-424	2.04	6.1	15.444

3	FW_60_1	1.05	356	bnlg2295	umc1124	332-380	2.14	6.5	-15.819

4	FW_60_2	4.04	144	gsy4	mmc0471	140-160	2.02	6.1	-11.272

5	FW_60_3	8.09	504	Umc2052	umc1384	488-504	2.26	6.9	14.543

	*cumulated*							***19.5***	

6	FW_80_1	1.04	336	Bnlg1811	dupssr26	308-364	2.58*	7.5	-16.785

7	FW_80_2	1.08	584	Umc83C	mmc0041	576-592	3.36***	9.7	-18.124

8	FW_80_3	2.02	80	mmc0111	gsy53b	48-92	2.17	6.4	15.758

9	FW_80_4	4.04	156	mmc0471	umc1088	144-188	2.29	6.7	-13.621

10	FW_80_5	8.09	496	Umc2052	umc1384	484-504	2.73*	8.1	21.382

	*cumulated*							***38.4***	

11	DW_30_1	2.08	484	Umc1536	umc1049	468-496	2.2	6.5	-2.717

12	DW_30_2	5.01	60	mmc0151	umc147a	44-76	2.36	7	2.939

13	DW_30_3	9.07	480	gsy330	bnlg1129	460-492	4.09****	11.8	4.186

	*cumulated*							***25.3***	

14	DW_40_1	5.05	392	Umc1822	umc54	372-420	2.1	**6.3**	5.466

15	DW_60_1	1.04	340	dupssr26	bnlg2295	332-364	4.52****	13.1	-9.324

16	DW_60_2	2.08	536	dupssr24	umc1464	512-548	2.97**	8.8	-6.879

17	DW_60_3	5.04	344	gsy34	umc1221	324-364	2.14	6.4	-7.937

18	DW_60_4	5.07	536	Umc1537	umc68	508-552	2.34	7	7.869

19	DW_60_5	5.07	580	Umc68	bnlg118	568-592	3.33***	9.9	-7.540

20	DW_60_6	8.09	500	Umc2052	umc1384	484-504	2.5*	7.6	8.503

	*cumulated*							***52.8***	

21	DW_80_1	1.01	0	Bnlg1124	umc1354	0-20	2.08	6.2	7.824

22	DW_80_2	1.04	336	Bnlg1811	dupssr26	328-356	3.6***	10.3	-11.965

23	DW_80_3	1.08	584	Umc83C	mmc0041	572-596	2.03	6	-8.734

24	DW_80_4	5.04	324	Umc1110	bnl7_71	284-344	2.41	7	-9.851

25	DW_80_5	8.09	504	Umc2052	umc1384	488-504	2.46*	7.3	10.686

	*cumulated*							***36.8***	

26	%DW_30_1	4.04	136	phi096	gsy4	132-148	2.23	6.6	1.025

27	%DW_30_2	4.06	264	Bnlg1621	umc1329	236-292	2.01	6	-1.134

28	%DW_30_3	5.01	12	Bnlg1006	mmc0151	0-32	2.47*	7.3	-1.175

	*cumulated*							***19.9***	

29	%DW_60_1	1.01	32	Umc1354	bnlg1014	0-64	2	6	1.318

30	%DW_60_2	1.06	440	Umc1590	umc1335	420-484	2.13	6.4	1.197

31	%DW_60_3	3.02	52	Umc1892	bnlg1325	40-72	4.65****	13.5	1.750

32	%DW_60_4	5.02	128	Umc90	csu108	104-152	2.61*	7.8	1.573

33	%DW_60_5	6.05	308	Umc1020	bnlg1702	296-332	2.33	7	-0.944

	*cumulated*							***40.7***	

34	%DW_80_1	4.05	180	Bnlg1217	umc1511	172-208	2.24	6.6	1.051

35	%DW_80_2	5.01	52	mmc0151	umc147a	32-72	2.62*	7.6	1.294

36	%DW_80_3	5.07	528	Umc1537	umc68	508-544	2.35	6.9	-0.991

37	%DW_80_4	8.01	48	Bnlg1194	bnl9_11	32-76	2.85*	8.3	-1.221

38	%DW_80_5	9.04	220	csu147	umc38c	196-244	3.04**	8.8	1.625

39	%DW_80_6	9.07	464	Umc1137	gsy330	440-488	4.38****	12.4	1.438

	*cumulated*							***50.6***	

40	Water_30_1	4.03	116	Umc2039	phi096	76-144	2.25	6.7	-10.951

41	Water_30_2	7.03	216	Bnlg339	bnlg155	196-228	2.24	6.6	-9.338

	*cumulated*							**13.3**	

42	Water_60_1	1.06	432	Umc1590	umc1335	404-448	2.83*	8.4	-8.631

43	Water_60_2	3.02	40	Umc1892	bnlg1325	12-52	3.01**	9	-9.340

44	Water_60_3	4.04	148	mmc0471	umc1088	116-156	3.29***	9.7	-8.285

45	Water_60_4	6.05	308	Umc1020	bnlg1702	296-364	2.47*	7.4	6.476

46	Water_60_5	7.02	80	Umc1549	bnlg1792	68-100	2.04	6.2	-5.933

	*cumulated*							**40.7**	

47	Water_80_1	1.08	584	Umc83C	mmc0041	572-592	3.89***	11.1	-8.425

48	Water_80_2	2.06	372	Bnlg1138	umc1079	348-380	2.37*	6.9	6.296

49	Water_80_3	4.04	160	phi079	bnlg1937	144-172	3.71***	10.6	-7.492

50	Water_80_4	4.04	176	Bnlg1217	umc1511	172-188	3.42***	9.9	-7.515

51	Water_80_5	8.01	44	Bnlg1194	bnl9_11	28-64	2.29	6.7	7.106

	*cumulated*							***45.2***	

52	ABA_embryo_30_1	2.04	280	Bnlg1175		264-301	2.31	7.1	66.365

53	ABA_embryo_30_2	2.08	604	Bnlg1940		84-620	2.75*	8.4	63.745

54	ABA_embryo_30_3	7.05	364	csu17		48-388	2.06	6.4	-52.461

	*cumulated*							***21.9***	

55	ABA_embryo_40_1	1.11	820	Bnlg131	bnl6_32	804-836	2.32	7	33.212

56	ABA_embryo_40_2	2.04	288	Bnlg1175	umc1007	272-304	3.27***	9.6	50.724

57	ABA_embryo_40_3	5.07	528	Umc1537	umc68	504-544	2.53*	7.5	-35.741

	*cumulated*							***24.1***	

58	ABA_embryo_60_1	9.04	236	csu147	umc38c	212-264	2.01	**6.1**	-66.055

59	ABA_embryo_80_1	5.02	120	Umc90	csu108	96-144	2.31	6.7	-71.183

60	ABA_embryo_80_2	7.03	224	Bnlg339	bnlg155	216-232	4.07****	11.5	-70.534

61	ABA_embryo_80_3	9.07	476	gsy330	bnlg1129	464-492	2.9**	8.4	-58.211

	*cumulated*							***26.6***	

62	ABA_endosperm_30_1	3.07	356	Bnlg197		344-388	2.53*	7.5	-8.566

63	ABA_endosperm_30_2	7.02	136	Umc5b		124-148	2.91**	8.6	-10.838

64	ABA_endosperm_30_3	9.07	476	gsy330		460-492	3.93***	11.4	-10.131

	*cumulated*							***27.5***	

65	ABA_endosperm_40_1	2.04	296	Bnlg1175	umc1007	284-312	3.34***	9.8	5.957

66	ABA_endosperm_40_2	5.03	256	Umc43	umc1056	244-264	2.58*	7.7	-4.642

67	ABA_endosperm_40_3	10.04	216	gsy87	bnlg1074	200-236	2.71*	8	4.586

	*cumulated*							***25.5***	

68	ABA_endosperm_60_1	3.03	64	Bnlg1325	bnlg1523	40-100	2.24	6.7	4.502

69	ABA_endosperm_60_2	4.10	556	Umc2011	bnlg1337	540-572	2.02	6.1	-4.458

	*cumulated*							***12.8***	

70	ABA_endosperm_80_1	7.03	244	csu37a	bnlg1666	236-288	2.02	6	1.232

71	ABA_endosperm_80_2	9.07	452	dupssr29	umc1137	432-468	2.23	6.5	-1.202

	*cumulated*							***12.5***	

72	Slope_1	2.04	316	csu143	bnlg371	306-336	2.92**	8.5	0.002

73	Slope_2	2.06	376	Bnlg1138	umc1079	364-40	2.61*	7.6	0.002

74	Slope_3	3.04	220	csu30B	dupssr23	212-244	2.07	6.1	-0.002

75	Slope_4	4.03	80	nc004	umc2039	48-92	2.31	6.8	-0.002

	*cumulated*							***29***	

76	Rate_30_40_1	2.06	404	Umc1079	umc139L	388-424	2.54*	7.6	0.013

77	Rate_30_40_2	7.05	364	csu17	bnl16_06	352-380	3.22***	9.6	0.017

	*cumulated*							***17.2***	

78	Rate_40_60_1	3.03	64	Bnlg1325	bnlg1523	48-72	3.51***	**10.5**	0.008

Position in pcM on the LHRF_F_3:4__1201 map of the segregating population. ABA_embryo: ABA in embryo (pg/DW); ABA_endosperm: ABA in endosperm (pg/DW); DW: kernel dry matter weight (mg/kernel); FW: kernel fresh matter weight (mg/kernel); %DW = DW/FW× 100; Water: kernel water content (mg/kernel); Rate = (Water/FW × 100)/(thermal time interval); Slope: regression line slope of (Water/FW × 100) as a function of thermal time. The genomewide risk, *P*, (absence of any QTL in the genome) was computed by classical permutation for a given LOD score (see Methods) and reported after the LOD value: * *P *< 0.25, ** *P *< 0.10, *** *P *< 0.05 and **** *P *< 0.01. The individual significance level (risk of absence of a QTL at a specified locus, not taking in account the multiple comparisons at other loci) is 0.001 for a 2.45 LOD score.

### QTLs for kernel desiccation and ABA content

**Figure 2:**
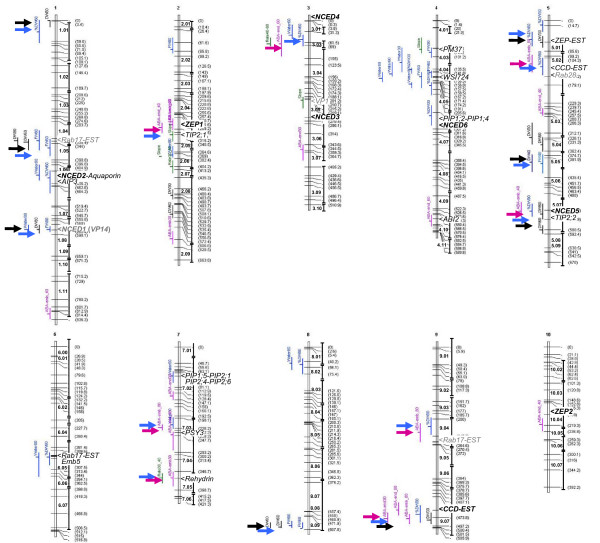
**QTLs and candidate gene map**. The LHRF_ F_3:4_segregating population was used. Bins are shown on the right of chromosomes. Distances are in pcM in the LHRF_ F_3:4_map (LHREF3_1201). QTLs for desiccation (blue), desiccation rate (green), growth (black) and ABA content (pink) are on the left of the chromosomes (Tables 2 and 3). The confidence intervals of the QTLs are indicated by vertical bars. Arrows highlight colocations between desiccation QTLs (blue) and growth QTLs (black) and between desiccation QTLs and ABA QTLs (pink). Gene locations in the QTL confidence interval are indicated close to their corresponding QTLs. Gene codes are detailed in Tables 3 and 4. Genes in bold were mapped by PCR in this study (Additional file 2). The others were mapped by Génoplante http://urgi.versailles.inra.fr/GnpMap/. Genes involved in kernel drying and located in the vicinity of the QTL confidence interval, are indicated in grey. ABA-emb: ABA in embryo (pg/DW); ABA-end: ABA in endosperm (pg/DW); DW: kernel dry matter weight (mg/kernel); FW: kernel fresh matter weight (mg/kernel); %DW = DW/FW× 100; Water: kernel water content (mg/kernel); Rate = (Water/FW × 100)/(thermal time interval); Slope: regression line slope of (Water/FW × 100) as a function of thermal time.

QTLs were searched at 30, 40, 60 and 80 DAP for three traits related to desiccation (Water, FW and %DW, blue in Fig. 2), for one related to growth (DW, black in Fig. 2) and for ABA content either in embryo or endosperm (pink in Fig. 2). In addition, desiccation rate was evaluated by five traits, the slope of the FW decrease from 30 to 80 DAP vs. thermal time and the slope between each of the sampling dates (30 to 40, 40 to 60, 60 to 80 and 30 to 80 DAP, green in Fig. 2). A total of 78 QTLs were detected for 25 of the 29 examined traits (Table 2). Out of the 29 traits analyzed, 13 displayed at least one QTL with a genomewide *P *value below 5%, confirming unambiguously that their variation was unlikely due to environmental effect, but rather to a genetic effect (Table 2). On average, two to three QTLs were detected for each trait, their sum explaining from 6.1 (ABA_embryo_60_1) to 52.8% (DW_60) of the phenotypic variation albeit rather low LOD scores (2 to 4.6). The fact that each trait was measured on two plants per F_3:4 _family from the same plot (unreplicated design) might explain these moderate effects. The experimental design did not allow for a correct estimation of heritabilities but one may assume they were low. In order to evaluate the uncertainties due to low heritability, genomewide risk was calculated for each QTL (see asterisks in Tables 2 and 3). As classically observed, the QTLs for different traits tend to be grouped in clusters that were not evenly distributed in the genome (Fig. 2). In nearly each cluster one or several QTLs were detected with a genomewide risk below 5% (e.g. bins 1.04, 1.08, 2.05, 3.02, 4.04, 5.07, 7.03, 9.07). Forty three QTLs were related to kernel desiccation, 15 to kernel weight and 20 to ABA concentration (Fig. 2, Table 2).

**Table 3:** Colocation between QTLs and candidate genes related to kernel desiccation and ABA biosynthesis

QTL name	Class	Bin	Position	From-to	Candidate gene
DW_80_2 ***	Growth	1.04		250-273	*Rab17-EST *(241)
				
FW_80_1 *	Desiccation		255	245-277	

Water_60_1 *	Desiccation		333	305-344	***NCED2 ***(310)
			
%DW_60_2	Desiccation	1.06	338	323-386	*Aquaporin *(314) *AIP3 *(323)

DW_80_3	Desiccation		460	455-464	
			
FW_80_2 ***	Desiccation	1.08-1.09	460	457-463	***NCED1 = Vp14 ***(465)
			
Water_80_1 ***	Desiccation		460	455-463	

ABA_embryo_30_1	ABA content		222	215-232	***ZEP1 ***(227)
			
ABA_embryo_40_2 ***	ABA content	2.04	226	219-234	
			
ABA_endosperm_40_1 ***	ABA content		230	224-237	*TIP2;1 *(236)
			
Slope_1 **	Desiccation rate		237	235-241	

Slope_3	Desiccation rate	3.05	220	212-244	*Vp1 *(209)

Slope_4	Desiccation rate		82	66-97	*PM37 *(82)
			
FW_30_1 *	Desiccation	4.03	92	79-109	

FW_80_4	Desiccation		130	124-142	*WSI724 *(132)
			
Water_80_3 ***	Desiccation	4.04-4.05	132	124-132	

%DW_30_2	Desiccation	4.06	173	161-189	*PIP1;2 PIP1;4 *(161)

ABA_endosperm_60_2	ABA content	4.10	340	329-346	ABI2 (334)

%DW_80_2 *	Desiccation		44	33-58	*ZEP-EST*(41)
			
DW_30_2	Growth	5.01	48	40-61	

ABA_embryo_80_1	ABA content		96	77-115	*CCD-EST *(81)
			
%DW_60_4 *	Desiccation	5.02	102	83-122	*Rab28 *(132)

%DW_80_3	Desiccation		312	304-322	
			
ABA_embryo_40_3 *	ABA content	5.07		302-322	***NCED5 ***(311)
			
DW_60_4	Growth		317	304-327	*TIP2;2 *(320)

%DW_60_5	Desiccation	6.05	148	136-175	*Rab17-EST *(138)
			
Water_60_4 **	Desiccation			136-201	*Emb5 *(138)

Water_60_5	Desiccation		167	163-173	*PIP1;5 *(164)
			
ABA_endosperm_30_2 **	ABA content	7.02	183	179-189	*PIP2;1 *(164) *PIP2;4 *(164) *PIP2;6 *(164)

ABA_endosperm_80_1	ABA content	7.03	244	236-288	*PSY3 *(241)

ABA_embryo_30_3	ABA content	7.05	383	365-402	*Rehydrin *(380)
				
Rate_30_40_2 ***	Desiccation rate			370-396	

%DW_80_5 **	Desiccation	9.04	125	108-140	*Rab17-EST *(150)
			
ABA_embryo_60_1	ABA content		135	120-149	

ABA_endosperm_80_2	ABA content	9.07	279	262-294	***CCD-EST ***(271)
			
%DW_80_6 ****	Desiccation		291	269-314	

To compare with gene positions, QTLs mapped on the LHRF_F_3:4 _segregating population (Additional file 1) were projected on the REFMAP050110 map [32] using Biomercator [34]. Distances on REFMAP050110 are expressed in pcM and are shown in parentheses for gene candidates. Genes in bold were mapped by PCR in this study (Tables 4 and Additional file 1). Genes involved in kernel drying and located in the vicinity of the QTL confidence interval, are indicated in grey. ABI2: ABA-insensitive protein phosphatase 2C 2; AIP3: ABI3-interacting protein 3; Emb5: Embryogenic-ABA-inducible LEA 5; PM37: seed maturation protein; PSY3: phytoene synthase 3; Vp1: Viviparous1; WSI724: dehydrin. The asterisks behind each QTL represent the genomewide risk *P *(for definition see legend of Table 2) with * *P *< 0.25, ** *P *< 0.10, *** *P *< 0.05 and **** *P *< 0.01.

The largest cluster on bin 4.04 only consisted of desiccation traits, whereas in the other clusters desiccation and growth traits were intermixed, which was expected due to the observed correlation between FW and DW at each DAP stage (Additional file 1). A focus on the 80 DAP stage at which maximum differences in drying were observed between both genotypes (Fig. 1A), showed that among the six %DW80 QTLs, a variable which mirrored moisture content, two colocated with FW80 and/or Water80 (bins 4.04 and 8.02) and three with QTLs for ABA content (bins 5.07, 9.04 and 9.07). The allele effect was consistent with the better drying performance of the F252 parent since four of the six %DW80 QTLs with a cumulated R^2 ^of 35.4% (total R^2^: 50.6%) presented a positive allele effect originating from F252.

QTLs for ABA content in embryo and endosperm were rarely colocated, which was somewhat predictable due to the poor correlation between ABA contents in the two tissues. By contrast, numerous colocations were observed between desiccation traits and ABA traits at eight loci (bins 2.04, 3.03, 5.02, 5.07, 7.03, 7.05, 9.04 and 9.07), illustrated by overlaps of their QTL confidence intervals (compare blue and pink arrows in Fig. 2).

### QTL and gene colocation

When clusters are composed of traits of different classes (desiccation, desiccation rate, growth, ABA content), QTL colocations raise the classical question of the existence of common genes which may control genetic variability of two or more classes at a single locus. A way to find candidate genes is to examine the list of reported genes which have been mapped in the QTL region. For this purpose, the QTLs mapped on the LHRF_F_3:4 _population (LHREF3_1201 map) were projected with Biomercator [34] on a reference map (REFMAP050110), which is based on the internationally used IBM population. The list of known or putative cDNAs provided by the data base in each QTL confidence interval was manually scanned to select functions related to water transfer (e.g. aquaporin), kernel maturation (e.g. LEA proteins) and ABA metabolism or regulation (e.g. ABA biosynthesis, ABA-responsive proteins and related transcription factors).

Relevant colocations (i.e. genes in the QTL confidence interval and functionally related to the trait) were observed on chromosomes 1, 2, 3, 4, 5, 6, 7 and 9 (Table 3). It has to be noted that most colocations of interest (Table 3) involved at least one QTL with a LOD score higher than 2.45, corresponding to an individual significance level below 0.001 and a genomewide level below 25%, including eight QTLs with a risk below 5% (see Methods). Desiccation trait clusters colocated with aquaporin ESTs (bins 1.06 and 4.06), maturation proteins (bins 1.04 and 4.03) and/or ABA-related genes (bins 1.06, 1.08-1.09, 3.05, 4.04-4.05, 5.01 and 6.05). Surprisingly, ABA biosynthetic *NCED1 *(*Vp14*; bin 1.08-1.09) and *NCED2 *(bin 1.06) genes did not colocate with QTLs for ABA content but rather with QTLs for desiccation. Nevertheless, colocations were identified for the ABA biosynthetic *ZEP1 *(bin 2.04) and *NCED5 *(bin 5.07) genes and QTLs for both desiccation and ABA content. Colocation of two *carotenoid cleavage dioxygenase *(*CCD*) ESTs was noted with clusters comprising desiccation and ABA traits (bins 5.02 and 9.07). However, although very close to *NCED *genes, *CCD *genes did not appear to be involved in ABA biosynthesis, the encoded enzymes being able to cleave carotenoids at 9,10 (9',10') bonds to generate multiple apocarotenoid products [35], whereas NCEDs cleaved carotenoids asymmetrically at positions 11-12 [36]. Another colocation involved the ABA_endosperm_80_1 QTL (bin 7.03) and the maize *phytoene synthase 3 *(*PSY3*) gene whose expression influences abiotic stress-induced root carotenogenesis [37]. Three other colocations involved QTLs for both desiccation and ABA content with genes encoding aquaporins (*TIP2;1 *on bin 2.04, *TIP2;2 *on bin 5.07 and *PIP1;5*, *PIP2;1*, *PIP2;4 *and *PIP2;6 *on bin 7.02) and LEA proteins (*Rab28 *on bin 5.02, *Rehydrin *on bin 7.05 and *Rab17 *on bin 9.04).

### Transcript expression of candidate genes related to water status, kernel maturation and ABA regulation during kernel desiccation

**Figure 3:**
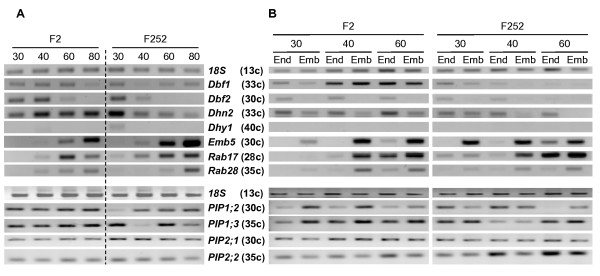
**Transcript profiling of candidate genes related to water transfer, kernel maturation and ABA regulation**. End point RT-PCR was performed on total RNA of the indicated tissues using gene-specific primers listed in Additional file 2. RNA quality and quantity were checked by total RNA loading on an agarose gel and ethidium bromide staining. The constitutively expressed 18 *S *rRNA gene was used as an internal control of RNA quantity. A, whole kernels without glumes at 30 to 80 DAP. B, Endosperms (End) and Embryos (Emb) at 30 to 60 DAP. The number of PCR cycles (end point) is indicated in brackets after the gene name.

As a first step to validate candidate genes, RT-PCR analysis was performed during kernel desiccation in both parental inbred lines for genes encoding LEA proteins, ABA-responsive transcription factors and aquaporins. In addition to parental differences, responses to desiccation might be classified into three categories: up-regulation, down-regulation and up-and-down-regulation (Fig. 3). Expression of LEA *Emb5 *and *Rab17 *(*Dhn1*) genes and dehydrin *Rab28 *gene increased during desiccation, especially in F252 at 60 and 80 DAP (Fig. 3A). Expression of *Dbf1 *and *Dbf2 *genes encoding transcription factors regulating the LEA *Rab17 *gene [38] diverged. *Dbf2 *expression clearly decreased over the time, whereas *Dbf1 *expression was still substantial at 80 DAP (Fig. 3A). The dehydrin *Dhy1 *gene was expressed at very low level. Nevertheless, its transcript levels clearly decreased in F252 genotype only, a pattern clearly established for the dehydrin *Dhn2 *gene (Fig. 3A). Furthermore, *Emb5*, *Rab17 *and *Rab28 *genes were more strongly expressed in embryo, whereas *Dhn2 *and *Dbf2 *genes were mostly expressed in endosperm (Fig. 3B). This pattern was conserved in both genotypes, although the magnitude of the expression was frequently different as previously noted in Fig. 3A.

Among the 12 analyzed *PIP *genes (Table 1), only *PIP1;2*, *PIP1;3*, *PIP2;1 *and *PIP2;2 *were repeatedly expressed in kernels (Fig. 3). *PIP1;2 *and *PIP1;3 *transcripts levels generally increased at later stages (Fig. 3A), but the time course was gene and genotype-dependent. Both genes showed stronger expression in embryo than in endosperm in F2 genotype, while no clear preference was seen in F252 genotype (Fig. 3B). In contrast, *PIP2;1 *and *PIP2;2 *expression was more or less stable during grain desiccation (Fig. 3A), with a preferential expression of *PIP2;2 *in older endosperm (Fig. 3B). The three TIPs tested (Additional file 2) had expression maximum in leaf and root tissue and only *TIP1;1 *was weakly expressed in kernels (data not shown).

### Molecular analysis of maize *NCED *and *ZEP *genes

**Figure 4:**
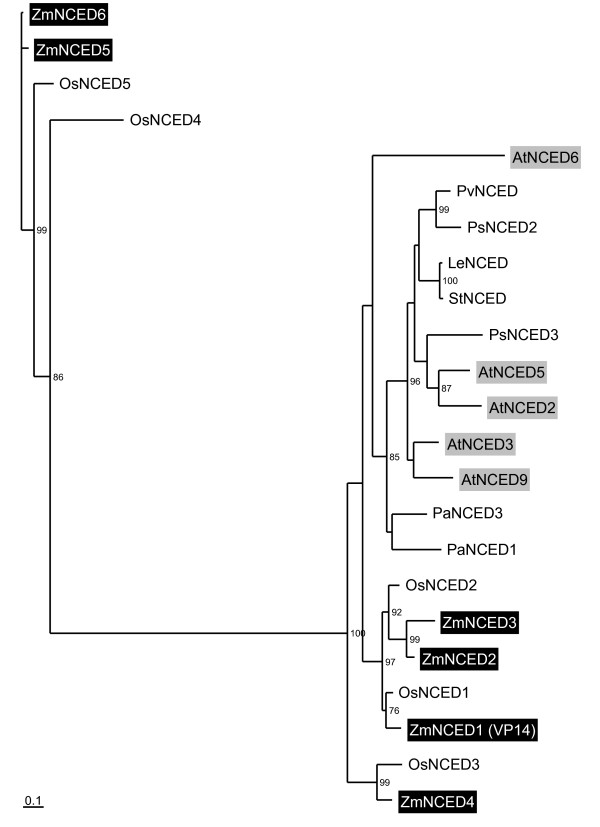
**Phylogenetic tree of NCED proteins**. The five novel maize NCED protein sequences were aligned with known NCED protein sequences [39,44,45]. In addition, four other NCED protein sequences were included in the alignment: OsNCED4 [GenBank:AAW21318.1] and OsNCED5 [GenBank:AAW21317.1] from rice and PsNCED2 [GenBank:BAC10550] and PsNCED3 [GenBank:BAC10551] from *Pisum sativum*. An unrooted tree ofthe NCED protein sequences was obtained using the maximum Likelihood method. Branch lengths are scaled proportional to substitution rate. Bootstrap values (percent) of 500 bootstrap replicates exceeding 70% are indicated above the supported branches. The Arabidopsis and maize NCEDs are highlighted by grey and dark boxes, respectively. Maize accession numbers are indicated in Table 4. Other NCEDs include: AtNCED2 [GenBank:NP_193569], AtNCED3 [GenBank:NP_188062], AtNCED5 [GenBank:NP_174302], AtNCED6 [GenBank:NP_189064] and AtNCED9 [GenBank:NP_177960] from Arabidopsis; LeNCED [GenBank:CAB10168] from *Lycopersicon esculentum*; OsNCED1 [GenBank:AAW21319.1], OsNCED2 [GenBank:AAW21321.1] and OsNCED3 [GenBank:AAW21320.1] from rice; PaNCED1 [GenBank:AF224672] and PaNCED3 [GenBank:AF224671] from *Phaseolus vulgaris*; StNCED [GenBank:CAB76920] from *Solanum tuberosum*.

One of the problems in analyzing accurately *NCED *and *ZEP *expression was the design of specific primers mainly because of insufficient knowledge of the actual number and sequence of *NCED *and *ZEP *genes in maize. Therefore, we first analyzed the public databases for the presence of putative *NCED *and *ZEP *coding sequences in maize. Nine different *NCED *loci had previously been identified with *NCED*-like EST sequences in the Génoplante programme, some ESTs mapping to several loci (e.g. E746, Table 4). To identify a maximum of *NCED *genes in maize, the search was extended to maize genomic contigs (The Institute for Genomic Research) and high throughput genomic sequences, which were matched with the EST sequences and used to design specific primers (Additional file 2) for DNA and cDNA amplification in lines F2 and F252. Focus on longest ESTs in the mixed cDNA/genomic DNA contigs allowed us to discard contigs with introns, which belonged to the *CCD *gene family [23] and to determine six maize *NCED *genes that could potentially encode NCED proteins (Table 4). Phylogenetic analysis performed with the deduced NCED amino acid sequences showed that the six maize proteins fell into four monocot clusters that were distinguishable from the eudicot cluster (Fig. 4). In contrast, close homologs for each of the maize *NCED *genes were found in rice. The two putative maize NCED5 and NCED6 proteins fell into a highly divergent cluster, including two maize and two rice NCED proteins. The orthologs of maize NCED1 (VP14) and NCED4 proteins were rice NCED1 and NCED3 proteins, respectively. Additionally, maize NCED2 and NCED3 proteins appeared as a pair of paralogs that was related to rice NCED2, whereas maize NCED5 and NCED6 proteins appeared as a pair of paralogs that was related to rice NCED5. *NCED1 *(*Vp14*), *NCED2*, *NCED3*, *NCED4 *and *NCED5 *genes were mapped on the LHRF mapping panel at five different loci (bins 1.08-1.09, 1.05-1.06, 3.05-3.06, 3.00 and 5.06-5.07) (Fig. 2, Table 4). *NCED6 *gene was mapped on the IBM mapping panel because of no polymorphism between F2 and F252 (bin 4.06; Fig. 2, Table 4). Only *NCED1, NCED2 *and *NCED5 *corresponded to previously mapped *NCED*-annotated ESTs, whereas no EST was detected for maize *NCED3, NCED4 *and *NCED6 *gene loci. Consistently, none of the identified *NCED *genes mapped at the *CCD *loci (bins 5.02 and 9.07; Table 3).

**Table 4:** Maize *NCED *and *ZEP *gene mapping and colocation with QTLs for desiccation and/or ABA content

Gene Name	EST	IGR ID^a^ HTGS ID^b^ yrGATE ID^c^	Map	Bin	**MM coord**.^d^	**Proj. coord**.^e^	Flanking markers	QTL colocation
*NCED1*	[GenBank:ZMU95953] E746	*Vp14* [GenBank:AC230016.2]	LHFR_Gnp2004	1.08- 1.09	504	466	bnlg1643- phi055	DW_80_3 FW_80_2 Water_80_1

*NCED2*	E746	AZM4_115740 [GenBank:AC217286.3]	LHFR_Gnp2004	1.05- 1.06	328	306	umc 1906- umc 67	Water_60_1 %DW_60_2

*NCED3*	No	AZM4_115695 [GenBank:AC199036.2]	LHFR_Gnp2004	3.05- 3.06	193	207	umc1501- dupssr23	No

*NCED4*	No	AZM4_114127 [GenBank:AC212820.3]	LHFR_Gnp2004	3.00	-108.7	-125	Before umc1746	No

*NCED5*	[GenBank:QCD5h12]	AZM4_50254 [GenBank:AC194862.3]	LHFR_Gnp2004	5.06- 5.07	364	298	phi087- umc1537	%DW_80_3 ABA_embryo_40_3 DW_60_4

*NCED6*	No	AZM4_50252 [GenBank:AC190588.3]	REFMAP050110	4.06	177	177	bnlg 1621- umc 66a	%DW_30_2

*ZEP1*	QAG5c10 [GenBank:AI977858]	AZM5_13314 [GenBank:AC194845.3] yrGATE_Zm2 gZEP1	REFMAP050110	2.04	221	221	bnlg1018- bnlg166	ABA_embryo_30_1 ABA_embryo_40_2 ABA_endosperm_40_1 Slope_1

*ZEP2*	QAG5c10	AZM5_13312 [GenBank:AC206194.3] yrGATE_Zm10 gZEP2	REFMAP050110	10.04- 10.05	94.5	94.5	bnlg1526- umc259	No

^a^The Institute for Genomic Research.

^b^HTGS: the High Throughput Genomic Sequences.

^c^yrGATE: Your Gene Structure Annotation Tool for Eukaryotes.

^d^Map coordinate computed with MapMaker using 'RI self Haldane' options.

^e^Map coordionate on REFMAP050110 obtained by homothetic projection with BioMercator [30].

Mapping of candidate genes was performed on the LHRF_Gnp2004 population derived from F2xF252 crossing, except for maize *NCED6*, *ZEP1 *and *ZEP2 *genes which were mapped on the IBM population (REFMAP050110 map) because of no polymorphism between F2 and F252 lines. QTL codes are detailed in Table 3.

Four *ZEP*-annotated ESTs had previously been mapped at bins 2.02-2.03, 4.09-4.11 and 5.01 (Génoplante data base, http://urgi.versailles.inra.fr/GnpMap, data not shown) in maize. Nevertheless, only two maize *ZEP *genes, *ZEP1 *(yrGATE_Zm2 gZEP1) and *ZEP2 *(yrGATE_Zm10 gZEP2), were ascertained from our data mining and PCR fragment joining (Additional file 3), in agreement with a recent report [37], and mapped on IBM mapping panel at bins 2.04 and 10.04-10.05, respectively (Fig. 2, Table 4).

### Transcript expression of maize *NCED *and *ZEP *genes during kernel desiccation

Colocations were identified for *NCED5 *and *ZEP1 *loci and QTLs for both desiccation and ABA traits, suggesting that these ABA biosynthetic genes are potential candidates (Fig. 2, Tables 3 and 4). Interestingly, *NCED1 *(*Vp14*), *NCED2 *and *NCED6 *genes mapped at loci colocating with QTLs for desiccation only (Fig. 2, Tables 3 and 4). Therefore, transcript levels of maize *NCED *and *ZEP *genes were quantified by quantitative RT-PCR (qRT-PCR) in embryo and endosperm from both parental lines at 40 and 60 DAP, ABA content being at the highest level in embryo at these stages (Fig. 1D). *NCED4 *expression was not analyzed because it was not possible to design *NCED4*-specific primers of sufficient quality for qRT-PCR.

**Figure 5:**
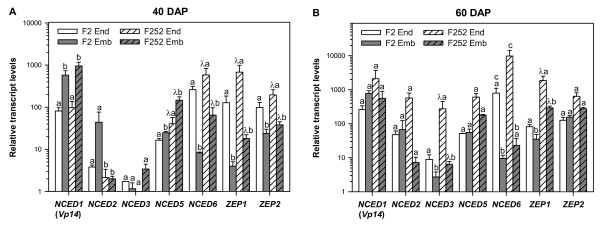
**Transcript profiling of maize *NCEDs *and *ZEPs *in kernel at 40 and 60 DAP**. qRT-PCR was performed on total RNA of the indicated tissues using gene-specific primers listed in Additional file 2. Gene codes are detailed in Table 4. RNA quality and quantity were checked by total RNA loading on an agarose gel and ethidium bromide staining. Transcript levels were normalized with the values obtained for the *Zeastar *gene, which was used as an internal reference gene, and are shown relative to *NCED3 *transcript levels in embryo at 40 DAP (the expression level of maize *NCED3 *in embryo at 40 DAP = 1). Values represent the mean of three biological replicates ± SE. When two samples show different letters above the bars, the difference between them is significant (*P *< 0.05). When both tissue and genotype effects were significant, a, b, λ a and λ b are indicated (see the Methods section and ANOVA in Additional file 4). End X: endosperm at x DAP; Emb X: embryo at X DAP.

*NCED1*, *NCED2*, *NCED3*, *NCED5 *and *NCED6 *transcripts were detected in all tissues analyzed, *NCED2 *and *NCED3 *being frequently expressed at a level close to the detection limit in most of the samples (Fig. 5). At 40 DAP, differential expression between embryo and endosperm was significant for the two mainly expressed *NCED *genes, *NCED1 *and *NCED6 *(Fig. 5A, Additional file 4) but the response was opposite since, in both genotypes, *NCED1 *transcript was present at the highest level in the embryo, while *NCED6 *was the most highly expressed transcript in the endosperm. *NCED5 *had a similar pattern to that of *NCED1*, although the magnitude of the embryo-endosperm difference in transcript level was lower than for *NCED1 *(Fig. 5A, Additional file 4). There was also a genotype effect since differential expression between F2 and F252 genotypes was significant for *NCED2*, *NCED5 *and *NCED6 *genes (Fig. 5A, Additional file 4). At 60 DAP, *NCED1*, *NCED2*, *NCED3 *and *NCED6 *transcript levels were higher in both genotypes than at 40 DAP (Fig. 5B). The difference between embryo and endosperm was no longer significant for *NCED1 *and *NCED5 *genes (Fig. 5B, Additional file 4). In contrast, expression was significantly higher in endosperm than in embryo for *NCED3 *and *NCED6 *genes (Fig. 5B, Additional file 3). Additionally, the genotype effect was significant for *NCED3*, its transcript level being lower in F2 genotype than in F252 genotype in both tissues (Fig. 5B, Additional file 4).

Regarding the *ZEP *genes, at 40 DAP, they were both mainly expressed in the endosperm rather than in the embryo (Fig. 5A, Additional file 4). There was also a genotype effect since expression of both *ZEP *genes was higher in the F252 genotype than in the F2 genotype in both tissues. At 60 DAP, *ZEP1 *preferential expression in the endosperm and higher expression in F252 were significant as observed at 40 DAP but magnitude of the effects was lower (Fig. 5B, Additional file 4). In contrast, there was no significant difference for *ZEP2 *expression between embryo and endosperm whatever the genotypes (Fig. 5B, Additional file 4).

## Discussion

### Relationship between kernel moisture and kernel weight

The observed genetic variability in the desiccation rate of maize inbred lines F2 and F252 and their offspring means that this trait is amenable for breeding purposes. The use of intermated recombinant inbred lines allows the reduction of QTL confidence interval to only a few pseudo cM (pcM, see Methods for equivalence between pcM and cM). On the basis of a map of roughly 6000 pcM and a genome size of 30 000 to 40 000 genes per maize genome, an estimate of 5 to 7 genes per pcM is reached. With the large progress in maize sequencing http://www.maizesequence.org, in maize genetic mapping, and syntenic relationships between maize and rice, a reasonable discrimination among candidate genes is possible in a given QTL confidence interval of a few pcM. In the case of physiological traits, candidate genes may be *a priori *selected for validation due to the knowledge of the process under study [30]. Although such a correlative approach does not establish direct causal relationships, it must be emphasized that the chance to find a random colocation between a QTL for the rather simple traits considered in this manuscript and a gene functionally related to the trait should be low. Nevertheless, the situation is different for more complex traits involving multiple developmental or metabolic pathways and consequently a much larger number of candidate genes. Using this approach, several candidates [39] were identified and then validated through molecular and/or genetic studies [40-42]. In the present case, obvious candidates for desiccation QTLs were LEA proteins and aquaporins, whereas obvious candidates for ABA QTLs were key enzymes of the ABA biosynthetic pathway, such as NCED and ZEP, ABA responsive genes and corresponding transcription factors.

Kernel moisture and kernel weight are non independent variables. It was recently shown that maximum kernel weight (MKW) may be predicted from maximum water content (MWC) occurring at 40 to 60 DAP in maize hybrids grown at three densities [1]. It was interpreted as meaning that maximum water content is an essential determinant of kernel volume, e.g. of sink capacity, which depends on early developmental events taking place during the lag phase. This MKW/MWC relationship was verified in the present parental lines and the LHRF_F_3:4 _population (data not shown), explaining the high correlations observed between FW and Water content and to a lesser extent FW and DW at each DAP stage. However, it has no influence on kernel drying as shown by the absence of correlation in the LHRF_F_3:4 _population between moisture content at harvest (%DW80) and final DW. The large observed variability in %DW, allowed detection of six QTLs explaining *ca*. 50% of the variance with most fast desiccation alleles contributed by parent 252. This was consistent with the higher desiccation rate of F252 parental line. Interrogation of Maize GDB http://www.maizegdb.org showed that the %DW60_4 and %DW80_5 QTLs mapped very close to earlier reported QTLs for kernel moisture on bins 5.02 and 9.04, respectively [43]. Comparison with the more recently reported grain moisture and drying rate QTLs [31] was difficult to assess because the map used was not anchored to any reference map and the QTL confidence intervals were very large. However, striking similarities were observed at bins 1.04, 1.08 and 5.01 when positions were estimated proportionally to the total length of each chromosome.

### Candidate gene mapping and colocations with QTLs

To the best of our knowledge, before the present study, QTLs for ABA content and water status have only been searched in leaves of plants submitted to water stress [39,44,45]. These QTLs were compared to those for ABA content and water related traits in kernel by projecting all of the QTLs on the reference map. The major *root-ABA1 *QTL identified near the RFLP marker *csu133 *on chromosome 2 (bin 2.04) [39,44,45] and affecting root architecture and grain yield in maize [46], did not colocate with the grain ABA QTLs detected in the same chromosome region (Fig. 2, Table 2). Nevertheless, striking similarities were observed in ten other regions (Table 5). Interestingly, among these ten regions, four (bins 1.08-1.09, 4.06, 7.01-7.02 and 7.03) were detected in all studies (one for kernel and three for leaves), the common candidates being *PIP *(in three regions out of four) and *NCED *(in two regions out of four) genes. The coincidence of QTL locations between a source (leaf) and a sink organ (kernel) strongly suggests that common genetic factors (genes) may control the trait at each locus. In this respect, our finding that *PIPs *are the most frequent candidate genes at the common loci is important as the data indicate that they are good candidates for the regulation of kernel moisture. Of particular interest is the observation that *PIP1;2 *and *PIP2;1 *are highly expressed isoforms, which are associated with water relations in both leaf [47] and kernel. By contrast, *NCED *candidates only appeared in relation to QTLs for leaf ABA content and not to QTLs for kernel ABA content.

**Table 5:** Common QTL locations for traits related to desiccation and ABA content in leaf and kernel

					QTLs from leaf		
					
Bin	Locus or marker	QTL position (pcM) on REFMAP050110	Candidate gene	QTLs from kernel	**Lebreton et al **[39,44,45].	**Tuberosa et al **[39,44,45].	**Pelleschi et al **[39,44,45].
1.06	bnl5.59	305-344	***NCED2*** *Aquaporin*	Water_60_1	ABAL		RWC, ABAX

1.08-1.09	umc39c	455-463	***NCED1***	DW_80_3, FW_80_2 Water_80_1	ΨL	ABAL	RWC, ABAX

3.05	umc10 gsy406	212-244	*Vp1*	Slope_3	ABAX		RWC ΨL

3.06	Gsy224	210-220	***NCED3***				ABAL

4.03-4.04	Gsy431	89 (66-97)	*PM37*	Slope_4			RWC

4.05-4.06	umc66	161-189	*PIP1;2, PIP1;4* ***NCED6***	%DW_30	ABAL	ABAL	ABAL, ΨL ABAX

7.01-7.02	gsy113 umc116	163-173 179-189	*PIP1;5, PIP2;1, PIP2;4, PIP2;6*	Water_60_5 ABA_endosperm_30_2	stomatal conductance	ABAL	ABAL ABAX

7.03	umc110	234-244 253-312	*PSY3*	ABA_embryo_80_2 ABA_endosperm_80_1	stomatal conductance	ABAL	RWC

9.04	umc114	108-149	*Rab17-EST*	%DW_80_5 ABA_embryo_60_1	ABAL		ΨL

9.07	gsy330 umc94	262-314	***CCD-EST***	%DW80_6 ABA_endosperm_80_2			ABAX

For kernel, the LHRF_F_3:4 _segregating population derived from F2 × F252 crossing. For leaf, populations derived from Polj17 × F2 crossing [39,44,45], high ABA × low ABA lines [39,44,45] and F2 × MBS847 RILs [39,44,45]. Plants were submitted to water deprivation at comparable young stage in [39,44,45] and [39,44,45]. In [39,44,45], ABA concentration was determined in leaf samples from stressed plants irrigated with 50% normal rainfall during stem elongation and before anthesis. Gene codes are detailed in Tables 3 and 4. Genes in bold were mapped by PCR in this study (Table 4 and Additional file 2). Genes involved in kernel drying and located in the vicinity of the QTL confidence interval, are indicated in grey. ABA_embryo: [ABA] in embryo; ABA_endosperm: [ABA] in endosperm; ABAL: leaf [ABA] ABAX: xylem [ABA]; DW: kernel dry matter weight (mg/kernel); FW: kernel fresh matter weight (mg/kernel); %DW = DW/FW× 100; Slope: regression line slope of (Water/FW × 100) as a function of thermal time; RWC: relative water content; Water: kernel water content (mg/kernel); ΨL: leaf water potential.

In addition to the above mentioned *PIP *and *NCED *genes, our kernel specific QTLs revealed other candidates such as the *ABI3-interacting protein 3 *(*AIP3*) on chromosome 1; *ZEP1 *and *TIP2;1 *on chromosome 2; *Viviparous1 *(*Vp1*) on chromosome 3; the *seed maturation protein 37 *(*PM37*), the dehydrin *WSI724 *and the *ABA-insensitive protein phosphatase 2C 2 *(*ABI2*) on chromosome 4; *Rab28*, *NCED5 *and *TIP2;2 *on chromosome 5; *Rehydrin *and *PSY3 *on chromosome 7; *Rab17 *and *CCD-EST *on chromosome 9 (Tables 3 and 4). The transcript patterns of *LEA*, *aquaporin*, *NCED *and *ZEP *genes during kernel maturation and desiccation provided further help for the identification of potential candidates. Regarding the desiccation traits, the consistency of *Rab17*, *Rab28 *and *Emb5 *genes as candidates was supported by the fact that their transcript levels increased from 40 to 80 DAP and was stronger in F252 than in F2, which was consistent with the positive effect of F252 allele on desiccation. It is also notable that ABA-responsive complex 3 found in *Rab28 *promoter sequence [48] is transactivated by VP1, which colocates with Slope_3 QTL. The situation was not as clear for the aquaporins, although some *PIP1 *and *PIP2 *were very frequently associated with QTLs for desiccation and ABA content and *PIP1 *gene expression increased by the end of the maturation phase. We suspect that specificity problem linked to the high sequence similarity between aquaporins led to cross-hybridization during RFLP-based mapping and/or non-specific amplifications in RT-PCR experiments, because primers were designed from the known 3' UTR B73 sequences, which likely differ from that of F2 and F252. We are confident that the emerging high throughput sequencing applied to individual lines like F2 and F252 will give us the necessary tools to obtain clear-cut answers in the future.

### Mapping, colocation and expression studies of the maize *NCED *and *ZEP *gene families

Effort was made to determine the number of *NCED *and *ZEP *genes in the maize genome, to map the genes on the LHRF mapping panel and to determine their expression patterns in the kernel of both maize parental inbred lines. The available data at the beginning of this work were (1) the *NCED1 *(*Vp14*) genomic sequence from B73, (2) the map positions of nine loci identified with a *NCED1 *RFLP probe or similarly annotated probes and (3) the mapping of four partial *ZEP *cDNAs at six loci. *In silico *analysis of genomic sequences coupled with PCR verification using genomic DNA and cDNA to confirm the contigs' physical existence and expression of sequence contigs, led to the identification of six *NCED *and two *ZEP *genes in maize. These figures remain minimum estimates based on the available data, but they are consistent with other species, since five *NCED *genes and a single copy *ZEP *gene have been found in Arabidopsis and rice. The tree topology of NCED1-related proteins suggests that at least four maize duplications might have occurred, one of which followed by high divergence in the branch supporting more recent duplicate NCED5 and NCED6 proteins. Additionally, blast analysis of rice high throughput genomic sequences revealed that maize *ZEP1 *and *ZEP2 *genes were closer to each other than to the rice *ZEP *gene, suggesting a maize specific duplication (data not shown). Mapping of maize *NCED *genes led to the identification of six different loci, only three being common to the nine previously reported loci. This highlights the caution needed in the interpretation of colocations between a QTL and a locus when the candidate gene was mapped using RFLP probes. Similar situations were found with *ZEP *genes since among the six loci identified with four partial *ZEP*-like cDNAs, only two were confirmed in agreement with a recent report [37].

The comparison of the transcript expression profiles of five of the six identified maize *NCED *genes shed new light on the relative autonomy of the embryo and the endosperm compartments. Accordingly, the expression patterns were independent in the endosperm and the embryo whatever the gene tested, suggesting different regulatory mechanisms. This was consistent with the absence of any correlation between ABA content or its time course in endosperm and embryo. The most strongly expressed maize *NCED *transcripts were *NCED1 *(*Vp14*) and *NCED6 *in embryo and endosperm, respectively. The higher expression of *NCED1 *in the embryo may explain why the *vp14 *mutation produced both a viviparous phenotype (vivipary being a property of the embryo) and a kernel ABA deficiency (the embryo being far much richer in ABA than the endosperm). However, the *NCED1 *locus did not colocate with embryo ABA QTLs, although it colocated with leaf ABA QTLs. A way to interpret this inconsistency in the kernel would be to assume that NCED1 is not limiting or provides a coarse control. The plausible existence of such a coarse control is consistent with the coincidence between maximum ABA accumulation and maximum *NCED1 *transcript level in the embryo at 60 DAP. An additional finer control might explain the genotype differences. It is noticeable that only *NCED5 *colocated with a QTL for ABA (ABAembryo40_3), this colocation being consistent with the fact that *NCED5 *transcript level was higher in the embryo than in the endosperm. Nevertheless, the low transcript level and the moderate R^2 ^associated with the QTL value might undermine the significance of this finding. Similarly to *NCED5*, and *NCED6*, *ZEP *transcript levels were significantly different in both genotypes and both tissues at 40 DAP. The expression of both maize *ZEP *genes was prevalent in the endosperm and higher in F252 line than in F2 line, *ZEP1 *being more strongly expressed than *ZEP2*. Interestingly, *ZEP1 *colocated with two QTLs for ABA content in the embryo at 30 and 40 DAP and one QTL for ABA content in endosperm at 40 DAP.

### Effect of ABA content on the genetic control of kernel desiccation

The potential role of ABA content on the genetic control of kernel desiccation was supported by numerous colocations between QTLs for both traits. The colocation of QTL for desiccation with *ABA-responsive *genes and *LEA *genes also known to be controlled by ABA provided further support, although it did not establish a triggering role of ABA on drying. To prove such a link, one would need to be able to ectopically manipulate ABA content in different parts of the kernel and examine the effect on desiccation. To this end, the genes controlling ABA content in grain must first be identified. *NCED *and *ZEP *genes which were the first to be shown to have a key role in ABA biosynthesis seemed good candidates prior to this study. However, among the 15 regions grouping the 20 ABA QTLs, only four QTLs colocated with *NCED5 *and *ZEP1 *loci. As previously mentioned, *NCED5 *was expressed at a lower level than *NCED1*, which did not colocate with any ABA QTLs, and both *ZEP1 *and *ZEP2 *genes were mainly expressed in endosperm in which ABA levels was much lower than in embryo. This can be interpreted as an indication that the control of ABA levels is unlikely to be determined by transcript expression. Post-transcriptional regulation should be checked by measuring NCED and ZEP protein levels as well as their enzyme activities. Other biosynthetic and/or other regulatory may also be considered. However, none of the newly reported regulator *Vp8*, *Vp10*/*Vp13 *and *Vp15 *genes [25-27] colocated with the presently detected ABA QTLs. It is noticeable that *PSY3 *gene colocated with ABA_endosperm_80_1 QTL on chromosome 7. Nevertheless, the low levels of *PSY3 *transcripts in maize endosperm [37] might undermine the significance of this finding. On the other hand, the genetic control of ABA content in leaf seems to present similarities to that in kernel as shown by the striking number of ABA QTLs of common loci between the two organs. Therefore, shared QTL loci and candidates merit special attention in future work, although the control is seemingly more complex in grain than in leaf.

## Conclusions

This study showed that kernel drying in field condition is genetically controlled. QTLs for kernel water status were frequently associated with QTLs for ABA content in embryo and/or endosperm, providing tools for marker assisted selection. In addition, striking colocations were found between the presently mapped QTLs for water status and ABA concentration in kernel and those previously reported in leaf for the same traits. Five novel *NCED *genes were identified and mapped. Phylogenetic analysis established homologies with rice genes. Unexpectedly, it was difficult to establish a causal relationship between the expression of individual members of the maize *NCED *and *ZEP *gene family and ABA QTL effect, although a few colocations between these genes and QTLs for ABA were detected. Among the other candidate genes mapped, colocations occurred for *aquaporins*, *LEA *and *ABA-responsive *genes and QTLs for kernel desiccation, indicating the potential interest of these genes for breeding and highlighting the necessity to validate them through transgenesis and association genetics.

## Methods

### Plant population and growing conditions

The population used was obtained by performing four generations of random intermating in the F_2 _population derived from the maize F2 × F252 hybrid. Random mating was performed by crossing two plants taken at random. Each plant being used only once, between 80 and 100 crosses were done at each generation to produce the next one. This was followed by one generation of selfing to produce a generation equivalent to F_3 _(LHRF_F_3_) in terms of genotypic frequencies. Each plant was again selfed to derive LHRF_F_3:4 _families (Y.F. Huang, D. Madur, V. Combes, C. L. Ky, P. Bertin, A. Charcosset, L. Moreau, in preparation). The parental lines and the whole LHRF_F_3:4 _population (322 families) were grown in the field at Gif-sur-Yvette, France, in April 2001, in rows (25 plants per rows, one row per family and two rows per parental line). Among the 322 grown families, 153 were sampled 120, 300, 400, 600 and 800 degree × day (cumulated average day temperature above a 6°C basis) after flowering for analyzing time course of desiccation and ABA content. At each stage, two plants per row were sampled for the LHRF_F_3:4 _families. For the parents, three plants in each of the two rows were sampled. We used a thermal scale to compensate for the difference in the dates of plant pollination between genotypes (from July 10th to August 1st, 2001). This scale corresponded to 12, 30, 40, 60 and 80 DAP for the year 2001, thus for simplification all the results were presented as DAP on a 2001 basis. At each date, 20 grains were sampled in the median part of the ear from two plants in each line. They were rapidly frozen in liquid nitrogen and stored in liquid nitrogen.

Statistical analysis of the data (mean, standard error and principal component analysis PCA) were performed using StatBox-Pro package (Grimmersoft, version 6.6, Issy Les Moulineaux, France)

### Kernel desiccation measurement

FW (mg/kernel) and DW (mg/kernel) were measured on five kernels and the four following variables were calculated: i) Water: absolute kernel water content = FW-DW (mg/kernel); ii) %DW: = DW/FW × 100, this variable mirrored relative water content; iii) Slope: regression line slope of (Water/FW × 100) as a function of thermal time between 30 and 80 DAP; iv) Rate = (Water/FW × 100)/(thermal time interval) between two sampling dates.

### ABA content measurement

Embryo and endosperm were separated from the pericarp and treated with two slightly different extraction protocols to optimise grinding efficiency. The embryos were ground with two stainless beads (3 mm diameter) at 20 Hz during 2 fold 30 s and the powder was lyophilized for 24 h. Then, 15 mg were placed in 200 μL distilled water and the slurry was agitated for 48 h at 4°C in the dark. This procedure prevented the formation of a lipid-powder mixture impeding ABA extraction. The whole kernels or the endosperms were lyophilized and ground with 2 stainless beads (7 mm diameter) at 20 Hz during 2 fold 30 s. Two or three aliquots of 250 mg were suspended in 700 μL distillated water for 48 h at 4°C in the dark. All samples were then centrifuged (12,000 *g*, 10 min) for clarification and the supernatants were used directly for radioimmuno assay (RIA) measurement as previously described. The monoclonal antibody used is only directed against the non-glycosylated ABA form [49]. Values were expressed as ABA content in pg per mg of FW, pg per mg of DW or pg per mg kernel water.

### QTL mapping

The map used for QTL detection was established using the whole population of 322 LHFR_F_3:4 _families (Y.F. Huang, D. Madur, V. Combes, C. L. Ky, P. Bertin, A. Charcosset, L. Moreau, in preparation). Briefly, leaves of each LHRF_F_3:4 _family (pool of 5 to 20 plants) were harvested to perform genomic DNA isolation, digestion and hybridization for RFLPs and amplified fragment length polymorphisms as previously described [50]. All the lines were characterized for 78 RFLP and 280 SSR markers. Genetic map was constructed using MAPMAKER 3.0b [51]. The genetic distances were calculated with Haldane mapping function. Because the LHFR_F_3:4 _population was regarded as a conventional F_3 _population (ignoring the generations of random intermating), those distances were not expressed in "true" cM and were referred to pcM. The linkage map included 358 markers with a total length of 5568.3 pcM and an average distance of 16 pcM between adjacent markers. By comparison with the map of a conventional F_3 _population derived from the same parental lines, the map expansion factor was estimated to be 2.63 on average over the whole genome (Y.F. Huang, D. Madur, V. Combes, C. L. Ky, P. Bertin, A. Charcosset, L. Moreau, in preparation).

The QTL detection was performed using composite interval mapping method implemented in the Plabqtl software [52]. Stepwise method was used for covariable identification using the "cov Select" option of the software. The LOD curve shape obtained with the "cov Select" option is strongly affected by the choice of cofactors, leading to artificially narrow peaks in the vicinity of covariate position, which is not appropriate to estimate QTL confidence intervals based on the one-LOD unit fall method. Therefore, confidence intervals, allele effect and individual R^2 ^of each QTL were estimated in a second step using the "cov-" option, which removes the markers located on the chromosome being analysed from the set of covariables initially selected by the "cov Select" option. The QTL detection threshold and the entry threshold for covariables were set respectively at LOD = 2 and *P *= 0.005.

To evaluate the risks associated with these threshold values, classical permutations were performed to determine the probability of the maximum of the test statistics under the null hypothesis (absence of any QTL in the genome). Maximum LOD values of 2.0, 2.36, 2.45, 2.88, 3.21 and 3.98 correspond to a genomewide type I risks of 0.5, 0.3, 0.25, 0.10, 0.05 and 0.01, respectively. The use of intermated recombinant inbred population increases the equivalent number of independent tests (over the genome) compared to conventional segregating populations and therefore increases the genomewide type I risk associated to a given LOD value. As such, a LOD 2 value can be considered as being permissive since it corresponds to a genomewide type I error risk of 0.5. Nevertheless, it corresponds to a low individual type I risk of 0.0025. Because we were looking for colocation between QTLs and candidate genes, even a colocation between a sub-significant QTL and a gene might be of interest especially if several QTL related to similar traits were also colocating. Therefore, we considered QTL with LOD > 2 in colocation analysis and carefully checked that each QTL region displayed at least one QTL with lower individual *P *values. The genomewide significance level of each QTL was indicated in Tables 2 and 3. The presently used unreplicated design was chosen to optimize the power of the QTL detection given the number of phenotypical observations, which are very labour intensive for the physiological traits of interest such as ABA content.

### Colocation between QTLs and candidate genes

Colocations between QTLs and candidate genes were based on manual search of the genes related to kernel desiccation and ABA biosynthesis in the confidence interval of each QTL. In order to have the most exhaustive list of mapped genes, the consensus REFMAP050110 (IBM_Gnp2004) framework map http://urgi.versailles.inra.fr/GnpMap was used [50]. It contains ESTs, genes and QTLs mapped in the French plant genomics programme 'Génoplante'. The QTLs detected in the LHRF_F_3:4 _population (LHREF3_1201 map) were projected on the REFMAP050110 using BioMercator software [34]. Data mining was performed using the tools set up by 'Génoplante' http://urgi.versailles.inra.fr/, which yields the list and genetic coordinates of the genes or ESTs reported in the vicinity of the QTLs. Because of the use of intermated recombinant inbred lines, appropriate conversion was performed to compute actual cM from map coordinates obtained with MapMaker [32].

### Maize *NCED *and *ZEP *gene mapping

Based on Arabidopsis and rice sequences, putative *NCED *and *ZEP *coding sequences in maize were identified by Blast searches of EST assemblies in The Gene Index data base http://compbio.dfci.harvard.edu/tgi/plant.html, assemblies of genomic DNA fragments at The Institute for Genomic Research http://www.tigr.org/plantProjects.shtml and genomic sequences from BAC clones http://www.ncbi.nlm.nih.gov/HTGS. The members of the maize *NCED *and *ZEP *gene families suggested from these combined *in silico *analyses were confirmed by sequencing of amplified cDNA and gDNA which specific primers. Maize *ZEP *genes were annotated using yrGATE at PlantGDB http://www.plantgdb.org/prj/yrGATE/, yrGATE_Zm2 gZEP1 is substantially the same as GRMZM2G127139_T01). The yrGATE_Zm10 gZEP2 annotation is incomplete since the last version of the maize HTGS database is not currently included. Therefore, both maize *ZEP1 *and *ZEP2 *genes are also shown in Additional file 3, which was based on the alignment of rice and maize *ZEP *genes and constructed using gbioseq http://www.bioinformatics.org/project/?group_id=94. The maize *NCED *and *ZEP *genes were then mapped on the LHRF or IBM mapping panels using the LHRF_Gnp2004 or REFMAP050110 (IBM_Gnp2004) framework map, respectively [50]. LHRF is an intermated recombinant inbred line population derived from the cross between maize F2 and F252 inbred lines. IBM is an intermated recombinant inbred line population derived from the cross between maize B73 and Mo17 inbred lines http://www.maizegdb.org. It was used in the absence of polymorphism between F2 and F252 [53]. Genotyping was PCR-based as previously described [50]. Primers used for mapping maize *ZEP *and *NCED *genes are shown in Additional file 2. Gene positions obtained on LHRF_Gnp2004 map were then projected on the REFMAP050110 map using BioMercator software [34].

### RT-PCR and qRT-PCR

Gene expression patterns using RT-PCR were determined as previously described [54]. In these end point analyses, the cycle number was adjusted to yield non-saturated bands for samples with low expression. Primers used for RT-PCR analyses are shown in Additional file 2. They were designed based on Gene Bank data http://www.ncbi.nlm.nih.gov/Genbank/ for aquaporins using the accession numbers provided in [11] and based on the maize EST assembly produced by Genoplante-Info and now housed at the URGI http://urgi.versailles.inra.fr/GnpSeq/ for *LEA*, *RAB *and other maturation-related genes.

For qRT-PCR, RNA isolation was carried out essentially as previously described [54]. Briefly, tissues were ground to powder under liquid nitrogen and transferred to a tube containing equal volumes of extraction buffer (200 mM Tris-HCl pH 9, 400 mM KCl, 200 mM sucrose, 35 mM MgCl_2_, 25 mM EGTA) and phenol/chloroform/isoamyl alcohol (pH 8) and vortexed for 30 s. The resulting supernatant collected after centrifugation (15,000 *g*, 10 min at 15°C) was re-extracted twice with phenol/chloroform/isoamyl alcohol and then precipitated 20 min at -80°C by addition of 1 M acetic acid (0.1 volume) and 100% ethanol (2.5 volumes). The RNA pellet collected after centrifugation (15,000 *g*, 30 min at 4°C) was washed with 3 M Na-acetate (pH 5.2) and re-suspended in water. A second acetic acid/ethanol precipitation was performed before final re-suspension in water. RNA was treated with RNase free DNase and the DNAse inactivated according to the instructions of the supplier (Ambion, Applied Biosystems, Foster City, CA, USA). RNA was quantified in a spectrophotometer at 260 nm and its quality was assessed by gel analysis and 260/280 and 260/230 nm spectrophotometric ratios. Total RNA (5 μg) was used as a template to synthesize cDNA using 0.5 μg oligo dT primers (Invitrogen, Breda, The Netherlands), 100 units of SuperScript II (Invitrogen) and 40 units of recombinant Rnasin ribonuclease inhibitor (Promega, Madison, Wisconsin, USA) in a final volume of 20 μl.

Quantitative real-time PCR was performed with 10 μl of 1:50 (v/v) dilution of the first cDNA strands synthesized as described above using the 7500HT real-time PCR system (Applied Biosystems, Foster City, CA, USA) and the SYBR green PCR master mix (Applied Biosystems) according to the manufacturer's instructions. Each reaction was performed in duplicate and the real-time experiment was repeated two times. The absence of genomic DNA and primer dimers was confirmed by analysis of RT-minus and water control samples and by examination of dissociation curves. The change in fluorescence for each sample was analyzed using 7500 real-time PCR system sequence detection software 1.3.1 (Applied Biosystems). Primer sequences were designed using Primer Express 2.0 (Applied Biosystems). PCR products for candidate genes were sequenced to confirm their identity. Genes and primers used for qRT-PCR analyses are shown in Tables 4 and Additional file 2. Transcript levels were normalized with the values obtained for the housekeeping *Zeastar *gene (accession number AC196679), which was used as an internal reference gene. The fold change of transcript abundance of candidate genes was first calculated as 2^-ΔCt^, where ΔCt is the number of PCR cycles required to reach the log phase of amplification for the target gene minus the same measure for *Zeastar*. Transcript abundance of maize *NCED3 *in embryo at 40 DAP was adjusted to 100% and fold changes of transcripts from other genes were normalized via comparison with that of maize *NCED3*. A Log transformation of the expression data was then performed because the standard error values showed that the standard error increased in proportion to the treatment. Log-transformed values were used for two-way ANOVA analyses, using R software version 2.8.1. A F-test for interactions was performed to determine whether the additive model could be retained [55,56]. This additive model corresponds to a multiplicative model for the initial non-transformed values, with a, b, λa and λb being the four values of the multiplicative model. When the additive model could not be retained, the Bonferroni method was applied for pairwise comparisons (see Additional file 3 for calculations). The significance was placed at a 0.05 level.

### Sequence alignment and phylogenetic analysis

Amino acid sequences of open reading frames were initially aligned using ClustalW [57] with the BioEdit Sequence Alignment Editor 4.8.8 [58]. For the phylogenetic analysis, the N-terminal portions of the proteins were omitted because of the difficulty of confidently assessing primary homology among these sequences. After the N-terminal regions, the NCED sequences share high degrees of similarity. These regions were therefore included in the phylogenetic analyses which were performed using the maximum Likelihood method with PHYML v2.4.4 [59]. JTT was used as the substitution model with four substitution rate categories, the gamma parameter being estimated from the data. Node support was assessed through 500 bootstraps. A dendrogram was constructed using TreeView 1.5.2 http://taxonomy.zoology.gla.ac.uk/rod/rod.html.

## Authors' contributions

VC planned and carried out the sample collection from the field, set up and performed RIA for ABA quantification, carried out QTL detection, managed interaction with the Génoplante data base, and did preliminary molecular analysis for qRT-PCR; CR, performed *in silico *search of maize *ZEPs*, primer design and sequencing for confirming *in silico *analyses, applying to qRT-PCR and mapping analyses of *NCED *and *ZEP *gene families; LM, provided expertise in the use of intermated recombinant inbred lines and performed the statistical analysis for QTL detection with VC; AR participated in sample collection, in setting up and carrying out high throughput RIA for ABA quantification and in molecular analysis; AM^1,2 ^contributed to RIA and provided her expertise in aquaporin by designing specific primers; AM^5,6 ^carried out the RT-PCR; MF performed *NCED *and *ZEP *gene mapping by PCR; AC conceived the intermated recombinant inbred line population and participated in the experimental design; CT participated in the experimental design, in planning and collecting the samples, she largely contributed to manuscript editing; PR managed the RT-PCR measurements and contributed to editing; SC performed *in silico *search of maize *NCED *genes, carried out the phylogenetic analysis, managed the qRT-PCR experiment and the corresponding statistical analysis, then, critically revised the manuscript; JLP conceived and coordinated the study, managed its design and drafted the manuscript. All authors read and approved the final manuscript.

## Supplementary Material

Additional file 1 **Pearson correlation coefficient between variables for significant values (*P *≤ 0.05)**. Abbreviations: ABAemb: ABA in embryo (pg/DW); ABAend: ABA in endosperm (pg/DW); ABAgrain: ABA in whole grain (pg/DW); DW: kernel dry matter weight (mg/kernel); FW: kernel fresh matter weight (mg/kernel); %DW = DW/FW × 100; Water: kernel water content (mg/kernel); Rate = (Water/FW × 100)/(thermal time interval); Slope: regression line slope of (Water/FW × 100) as a function of thermal time. Click here for file

Additional file 2 **Primers used for mapping, RT-PCR and qRT-PCR**. displaying primers used for mapping, RT-PCR and qRT-PCR. Click here for file

Additional file 3 **Identification of two *ZEP *genes in maize**. The rice *ZEP *gene (Os04 g0448900; black) was used in BLAST analysis to identify five putative homologs from maize (The Institute for Genomic Research ID OGAGIC79TC, AZM5_13314, AZM5_13316, AZM5_24223, AZM_13312 and AZM5_13315; light brown). Maize ESTs were found for all this maize genomic contigs, except for AZM5_13315. Sequencing of amplified gDNA and cDNA with specific primers showed that ZEP is encoded by only two genes in maize (deduced CDS indicated in blue). This was confirmed using the maize HTGS database (maize *ZEP1*, [GenBank:AC194845.3]; maize *ZEP2*, [GenBank:AC206194.3]; brown) and in agreement with recent reports [39,44,45]. We used yrGATE at PlantGDB http://www.plantgdb.org/prj/yrGATE to annotate both genes(maize *ZEP1*: yrGATE_Zm2 gZEP1; maize *ZEP2*: yrGATE_Zm10 gZEP2; yrGATE_Zm10 gZEP2 gene annotation is incomplete since the last version of the maize HTGS database is not currently included in the software). Click here for file

Additional file 4 **Analysis of variance table and Bonferroni method**. containing analysis of variance table and Bonferroni method. Click here for file
