# Supplementary material for: QTLs and candidate genes for desiccation and abscisic acid content in maize kernels
Source: BMC Plant Biol. 2010 Jan 4;10:2. doi: 10.1186/1471-2229-10-2 (PMC2826337; doi:10.1186/1471-2229-10-2)
Supplement: Additional file 1 — Pearson correlation coefficient between variables for significant values (P ≤ 0.05). Abbreviations: ABAemb: ABA in embryo (pg/DW); ABAend: ABA in endosperm (pg/DW); ABAgrain: ABA in whole grain (pg/DW); DW: kernel dry matter weight (mg/kernel); FW: kernel fresh matter weight (mg/kernel); %DW = DW/FW × 100; Water: kernel water content (mg/kernel); Rate = (Water/FW × 100)/(thermal time interval); Slope: regression line slope of (Water/FW × 100) as a function of thermal time. [file 1471-2229-10-2-S1.DOC]

# Additional files

Additional file 1 – Pearson correlation coefficient between variables for significant values (*P* _≤_ 0.05). (.doc, Arial, 5, simple)

ABAemb: ABA in embryo (pg/DW); ABAend: ABA in endosperm (pg/DW); ABAgrain: ABA in whole grain (pg/DW); DW: kernel dry matter weight (mg/kernel); FW: kernel fresh matter weight (mg/kernel); %DW= DW/FW×100; Water: kernel water content (mg/kernel); Rate = (Water/FWx100)/(thermal time interval); Slope: regression line slope of (Water/FWx100) as a function of thermal time.

|  | FW30 | FW40 | FW60 | FW80 | DW30 | DW40 | DW60 | DW80 | %DW30 | %DW40 | %DW60 | %DW80 | Water30 | Water40 | Water60 | Water80 | ABAend30 | ABAend40 | ABAend60 | ABAend80 | ABAgrain12 | ABAemb30 | ABAemb40 | ABAemb60 | ABAemb80 | Slope | Rate30_40 | Rate40_60 | Rate60_80 |
| --- | --- | --- | --- | --- | --- | --- | --- | --- | --- | --- | --- | --- | --- | --- | --- | --- | --- | --- | --- | --- | --- | --- | --- | --- | --- | --- | --- | --- | --- |
| FW30 | 1 | **0.17** | **0.38** | **0.30** | **0.69** | 0.12 | **0.30** | **0.29** | **-0.33** | -0.12 | **-0.24** | -0.13 | **0.97** | **0.18** | **0.40** | **0.27** |  |  |  |  |  |  |  |  |  |  | **0.19** |  |  |
| FW40 | **0.17** | 1 | **0.18** |  |  | **0.87** | 0.16 | 0.04 | -0.06 | **-0.39** | -0.08 | -0.03 | **0.16** | **0.96** | **0.18** |  |  | **-0.32** |  |  |  |  |  |  |  |  | **-0.19** | **0.29** |  |
| FW60 | **0.38** | **0.18** | 1 | **0.58** | **0.20** | 0.07 | **0.92** | **0.56** | **-0.27** | **-0.23** | **-0.28** | **-0.23** | **0.39** | **0.23** | **0.91** | **0.51** |  |  |  |  | **0.38** |  | **-0.25** |  |  |  |  |  |  |
| FW80 | **0.30** |  | **0.58** | 1 |  | 0.08 | **0.54** | **0.94** | **-0.26** | 0.04 | **-0.16** | **-0.48** | **0.33** |  | **0.52** | **0.91** |  |  |  |  | **0.32** | **-0.18** |  |  | **0.28** | **0.38** | **0.24** | **-0.19** | **-0.27** |
| DW30 | **0.69** |  | **0.20** |  | 1 | 0.12 | **0.21** | **0.18** | **0.44** | -0.04 | 0.01 | 0.13 | **0.49** |  | 0.16 |  | **-0.16** |  |  |  |  | **0.19** |  |  |  |  | **-0.39** |  |  |
| DW40 |  | **0.87** |  |  |  | 1 |  |  |  |  |  |  |  | **0.71** |  |  |  | **-0.33** |  |  |  |  |  |  |  |  |  |  |  |
| DW60 | **0.30** |  | **0.92** | **0.54** | **0.21** | 0.09 | 1 | **0.57** | -0.15 | -0.14 | 0.11 | -0.08 | **0.28** |  | **0.68** | **0.41** |  |  |  |  | **0.34** |  | **-0.28** | 0.03 | 0.06 | -0.05 | 0.01 | **0.21** | **-0.17** |
| DW80 | **0.29** |  | **0.56** | **0.94** | **0.18** | 0.09 | **0.57** | 1 | **-0.16** |  |  | **-0.16** | **0.29** |  | **0.45** | **0.71** |  |  |  |  | **0.34** |  |  |  | **0.22** |  | **0.18** |  |  |
| %DW30 | **-0.33** |  | **-0.27** | **-0.26** | **0.44** |  |  | **-0.16** | 1 | 0.14 | **0.34** | **0.33** | **-0.55** |  | **-0.36** | **-0.35** |  |  |  | **-0.29** | **-0.17** | **0.25** |  |  | **-0.21** |  | **-0.74** | **0.19** |  |
| %DW40 |  | **-0.39** | **-0.23** |  |  |  |  | 0.09 | 0.14 | 1 | **0.22** | 0.11 | -0.13 | **-0.62** | **-0.27** |  |  |  |  |  |  |  |  |  |  |  | **0.51** | **-0.61** |  |
| %DW60 | **-0.24** |  | **-0.28** | **-0.16** |  |  |  | -0.03 | **0.34** | **0.22** | 1 | **0.41** | **-0.29** | -0.14 | **-0.64** | **-0.30** |  |  |  |  |  |  |  | **0.23** |  | **-0.45** |  | **0.58** | **-0.37** |
| %DW80 |  |  | **-0.23** | **-0.48** |  |  |  | **-0.16** | **0.33** | 0.11 | **0.41** | 1 | **-0.21** |  | **-0.35** | **-0.80** |  |  |  | **-0.21** |  | **0.17** |  |  | **-0.30** | **-0.86** | **-0.23** | **0.27** | **0.64** |
| Water30 | **0.97** |  | **0.39** | **0.33** | **0.49** |  | **0.28** | **0.29** | **-0.55** | -0.13 | **-0.29** | **-0.21** | 1 | **0.18** | **0.43** | **0.32** |  |  |  | **0.18** |  |  |  |  |  |  | **0.36** |  |  |
| Water40 | **0.18** | **0.96** | **0.23** |  |  | **0.71** | **0.18** |  |  | **-0.62** |  |  | **0.18** | 1 | **0.24** |  |  |  |  |  | **0.17** |  |  |  |  |  | **-0.30** | **0.42** |  |
| Water60 | **0.40** | **0.18** | **0.91** | **0.52** |  |  | **0.68** | **0.45** | **-0.36** | **-0.27** | **-0.64** | **-0.35** | **0.43** | **0.24** | 1 | **0.53** |  |  |  | **0.19** | **0.36** |  | **-0.19** |  | 0.09 | **0.29** | 0.08 | **-0.26** |  |
| Water80 | **0.27** |  | **0.51** | **0.91** |  |  | **0.41** | **0.71** | **-0.35** |  | **-0.30** | **-0.80** | **0.32** |  | **0.53** | 1 |  |  |  | **0.22** | **0.25** | **-0.21** |  |  | **0.32** | **0.66** | **0.29** | **-0.25** | **-0.49** |
| ABAend30 |  |  |  |  |  |  |  |  |  |  |  |  |  |  |  |  | 1 |  |  |  |  |  |  | **0.23** |  | **0.16** |  |  |  |
| ABAend40 |  | **-0.32** |  |  |  | **-0.33** |  |  |  |  |  |  |  | **-0.29** |  |  |  | 1 | **0.17** |  |  |  |  |  |  |  |  |  |  |
| ABAend60 |  |  |  |  |  |  |  |  |  |  |  |  |  |  |  |  |  | **0.17** | 1 |  | -0.15 |  |  | **0.19** | 0.07 | -0.16 | 0.03 | **0.17** | -0.08 |
| ABAend80 |  |  |  | **0.16** |  |  |  |  | **-0.29** |  | -0.16 | **-0.21** | **0.18** |  | **0.19** | **0.22** |  |  |  | 1 |  |  |  |  | **0.23** | 0.14 | **0.21** | -0.11 | -0.07 |
| ABAgrain12 |  |  | **0.38** | **0.32** |  |  | **0.34** | **0.34** | **-0.17** |  |  |  |  | **0.17** | **0.36** | **0.25** |  |  |  |  |  |  |  |  |  |  |  |  |  |
| ABAemb30 |  |  |  |  | **0.19** |  |  |  | **0.25** |  |  | **0.17** |  |  |  | **-0.21** |  |  |  |  |  |  |  |  |  |  | **-0.18** | -0.02 | 0.10 |
| ABAemb40 |  |  |  |  |  |  | **-0.28** |  |  |  |  |  |  |  | **-0.19** |  |  |  |  |  |  |  | 1 | **0.23** |  |  |  |  |  |
| ABAemb60 |  |  |  |  |  |  |  |  |  |  | **0.23** |  |  |  |  |  | **0.23** |  | **0.19** |  |  |  | **0.23** | 1 |  |  |  |  |  |
| ABAemb80 |  |  |  | **0.28** |  |  |  | **0.22** | **-0.21** |  |  | **-0.30** |  |  |  | **0.32** |  |  |  | **0.23** |  |  |  |  | 1 | **0.19** | **0.19** | -0.10 | **-0.23** |
| Slope |  |  |  | **0.38** |  |  |  |  |  |  | **-0.45** | **-0.86** |  |  | **0.29** | **0.66** | **0.16** |  |  |  |  |  |  |  | **0.19** | 1 |  | **-0.40** | **-0.46** |
| Rate30_40 | **0.19** | **-0.19** |  | **0.24** | **-0.39** |  |  |  | **-0.74** | **0.51** |  | **-0.23** | **0.36** | **-0.30** | 0.08 | **0.29** |  |  |  | **0.21** |  | **-0.18** |  |  | **0.19** | 0.03 | 1 | **-0.54** |  |
| Rate40_60 |  | **0.29** |  | **-0.19** |  |  | **0.21** |  | **0.19** | **-0.61** | **0.58** | **0.27** |  | **0.42** | **-0.26** | **-0.25** |  |  | **0.17** |  |  | -0.02 |  |  |  | **-0.40** | **-0.54** | 1 | **-0.26** |
| Rate60_80 |  |  |  | **-0.27** |  |  | **-0.17** |  |  |  | **-0.37** | **0.64** |  |  |  | **-0.49** |  |  |  |  |  |  |  | **-0.22** | **-0.23** | **-0.46** | -0.12 | **-0.26** | 1 |
