# Supplementary material for: QTLs and candidate genes for desiccation and abscisic acid content in maize kernels
Source: BMC Plant Biol. 2010 Jan 4;10:2. doi: 10.1186/1471-2229-10-2 (PMC2826337; doi:10.1186/1471-2229-10-2)
Supplement: Additional file 2 — Primers used for mapping, RT-PCR and qRT-PCR. displaying primers used for mapping, RT-PCR and qRT-PCR. [file 1471-2229-10-2-S2.DOC]

### Additional file 2 - Primers used for mapping, RT-PCR and qRT-PCR. (.doc, Times, 10, simple)

| **Gene** | **Used for** | **Primer** | **Tm** | **Amplicon**  **(bp)** | **Sequence (5’->3’)** |
| --- | --- | --- | --- | --- | --- |
| ***18S*** | RT-PCR | STD5 | 59 | 151 | CCATCCCTCCGTAGTTAGCTTC |
|  |  | STD6 | 59 |  | CCTGTCGGCCAAGGCTATATAC |
| ***pAW109*** | RT-PCR | STD9 | 57 | 230 | CATGTCAAATTTCACTGCTTCATC |
|  |  | STD10 | 60 |  | TGACCACCCAGCCATCCTT |
| ***EMB5*** | RT-PCR | EMB5-279F | 62 | 130 | TGAGCACCATGCAGGAGTCC |
|  |  | EMB5-408R |  |  | ATTCCTGCAGCTACGTACTACATGC |
| ***Rab17 (dhn1)*** | RT-PCR | dhn1-603F | 60 | 152 | CCCATAAGTACAGTGGCTGTGCT |
|  |  | dhn1-754R |  |  | ACGTACAAATTCACCCCACAAGTA |
| ***Dhn2*** | RT-PCR | dhn2-624F | 62 | 104 | ACGAAGACTCAGACCCCACCA |
|  |  | dhn2-727R |  |  | GCGTCTTCCGGCTTCTTGT |
| ***Dhy1*** | RT-PCR | dhy1-278F | 60 | 301 | TACTACCCCATTTCTGCACCCTC |
|  |  | dhy1-578R |  |  | GCTTGTTTGCCCCTCTCGT |
| ***Dbf1*** | RT-PCR | dbf1-666F | 60 | 302 | ACAGTTGGATTTCAGCGAGGTT |
|  |  | dbf1-967R |  |  | AGACAGACCAGTACACCAATGCA |
| ***Dbf2*** | RT-PCR | dbf2-854F | 60 | 143 | AGGATGTGGTCAATGCGGA |
|  |  | dbf2-996R |  |  | GCGAGAAGCAAAACGAAGAAGA |
| ***Rab28*** | RT-PCR | rab28-360F | 60 | 150 | CAACGATCCAATCCACACTCAC |
|  |  | rab28-509R |  |  | GCTGCTCCTGGCTCATTCTC |
| ***PIP1;1*** | RT-PCR | ZmPIP 1-1 F | 60 | 110 | TACGTGCAGTTCCATTCCTCTT |
|  |  | ZmPIP 1-1 R |  |  | TTGCACATAACAGAGGCGC |
| ***PIP1;2*** | RT-PCR | ZmPIP 1-2 F | 58 | 166 | TGCTGCGCCGTTCTGTTAA |
|  |  | ZmPIP 1-2 R |  |  | CATCTGCCCTTTCTTGCCA |
| ***PIP1;3*** | RT-PCR | ZmPIP 1-3 F | 56 | 194 | TTGTCTGCCCACGCCTAGTTT |
|  |  | ZmPIP 1-3 R |  |  | CACGATCCCAACGCATAAAAG |
| ***PIP1;5*** | RT-PCR | ZmPIP 1-5 F | 58 | 309 | TACCAACAGCAACCCAAAAT |
|  |  | ZmPIP 1-5 R |  |  | ACTTCACCGTAGCAAAACCC |
| ***PIP1;6*** | RT-PCR | ZmPIP 1-6 F | 58 | 131 | GTCATACACGGAGTTCTGCTGC |
|  |  | ZmPIP 1-6 R |  |  | ATGACACATTCCGTGGATGC |
| ***PIP2;1*** | RT-PCR | ZmPIP 2-1 F | 61 | 256 | CTTCAGGAGCAACGCGTGA |
|  |  | ZmPIP 2-1 R |  |  | GACCATAAGAGCAGAGCGGA |
| ***PIP2;2*** | RT-PCR | ZmPIP 2-2 F | 58 | 133 | CTCGTCGGTCGCTTTTGTT |
|  |  | ZmPIP 2-2 R |  |  | CGATCCCTTGGCAGCAGATAT |
| ***PIP2;3*** | RT-PCR | ZmPIP 2-3 F | 57 | 105 | TCGATCCAAGTGTAGATACGTACG |
|  |  | ZmPIP 2-3 R |  |  | AAAACAGCACCAAGCGGAC |
| ***PIP2;4*** | RT-PCR | ZmPIP 2-4 F | 58 | 121 | CTCGCTCGCTGCTGTTTTC |
|  |  | ZmPIP 2-4 R |  |  | CGGATAAAAACTCACGCAATTG |
| ***PIP2;5*** | RT-PCR | ZmPIP 2-5 F | 62 | 248 | GCCTCCTTCAGCCGCTAG |
|  |  | ZmPIP 2-5 R |  |  | GGTCGACTCGTCGTACACGG |
| ***PIP2;6*** | RT-PCR | ZmPIP 2-6 F | 58 | 193 | TATTCAGCTCTTCCTCCTCCGC |
|  |  | ZmPIP 2-6 R |  |  | CACACCGCACACAAACTCACA |
| ***PIP2;7*** | RT-PCR | ZmPIP 2-7 F | 58 | 109 | TGGAACCGGCATTAACCCT |
|  |  | ZmPIP 2-7 R |  |  | AGCTCCAACAAACGGTCCAAC |
| ***TIP1;1*** | RT-PCR | ZmTIP 1-1 F | 60 | 234 | TACTAAAAGCCGAAGCCGAC |
|  |  | ZmTIP 1-1 R |  |  | TTACAGAAGCAAACGGGTCC |
| ***TIP1;2*** | RT-PCR | ZmTIP 1-2 F | 56 | 262 | CCACGGCAGCAGACTACTGA |
|  |  | ZmTIP 1-2 R |  |  | GTCAAATTGATGCTTTAATAG |
| ***TIP1;3*** | RT-PCR | ZmTIP 2-3 F | 58 | 238 | GCCAGCAGGAGTACCCATGA |
|  |  | ZmTIP 2-3 R |  |  | GAAACGCCGAACTGTGCA |
| ***NCED1 (Vp14)*** | mapping and qRT-PCR | NCED1d-F | 60 | 150 | AGTTGTTGTCACCCAGTCCAG |
|  |  | NCED1d-R |  |  | CACGCACCGATAGCCACA |
| ***NCED2*** | mapping | AZM740_F_85 | 55 | 227 | ACGCCGACGACGGGCGCCAGC |
|  |  | AZM740_R_312 |  |  | CCGTGCCCAGGTCCACTTTC |
| ***NCED2*** | qRT-PCR | AZM740_F_593 | 60 | 81 | TTGATTGCTTGGTTCTTTCTTTACG |
|  |  | AZM740_R_668 |  |  | CGAGGAGCAGAGCGATATGG |
| ***NCED3*** | mapping | AZM695_F_980 | 55 | 619 | TACCCTCCCGCGTGCCCTATG |
|  |  | AZM695_R_1599 |  |  | AGCCCTCTCAATCGTAATGTG |
| ***NCED3*** | qRT-PCR | AZM695_F_1418 | 60 | 78 | GTTGTTCACTCATGCCAAGCA |
|  |  | AZM695_R_1491 |  |  | GAGTGTGTACGAGAACCAAATGAAA |
| ***NCED4*** | mapping | AZM127_F_2661 | 55 | 140 | CGATCACGAACTCGACCAACT |
|  |  | AZM127_R_2800 |  |  | CCCTCTACTGCTCTAATAATGAACAAATT |
| ***NCED5*** | mapping | NCED2-54-2-F | 55 | 293 | GCCGCTGGATACTGAATAGATTGT |
|  |  | NCED2-54-3-R |  |  | AGGTCAACGGACGCTATTAGATCA |
| ***NCED5*** | qRT-PCR | NCED2-54-3-F | 60 | 85 | ACCGACGAGGCGTTCGA |
|  |  | NCED2-54-3-R |  |  | AGGTCAACGGACGCTATTAGATCA |
| ***NCED6*** | mapping | NCED2-52-2-F | 55 | 370 | TGTAGCACATGATTCCAGGAGAAA |
|  |  | NCED2-52-1-R |  |  | CCCTCGCACGTTTGACATG |
| ***NCED6*** | qRT-PCR | NCED2-52-2-F | 60 | 86 | TGTAGCACATGATTCCAGGAGAAA |
|  |  | NCED2-52-2-R |  |  | TGTGGCCTAGAGTTGTTCTTACTACAA |
| ***ZEP1*** | mapping | AZM5_13314_1825F | 53 | 255 | AAATCCTTGCTCGAGCAGTC |
|  |  | AZM5_13314_2080R |  |  | TTGACCATATTCCATCGGCG |
| ***ZEP1*** | qRT-PCR | AZM5_24223_225F | 60 | 67 | GAATCGGCAGCAACAGCAA |
|  |  | AZM5_24223_291R |  |  | GATGATAGTGGTGGTAGCCAGTGT |
| ***ZEP2*** | mapping | AZM5_13312_2743F | 55 | 1019 | TCAAGACTGGAACTCCTATG |
|  |  | AZM5_13312_3762R |  |  | ATCGTCATCGTCAAACCACC |
| ***ZEP2*** | qRT-PCR | AZM5_13312_2774F | 60 | 121 | CTCCTTGAGACGTTATGAGAAAGAGA |
|  |  | AZM5_13312_3086R |  |  | AGGTCCTAGACCAACACCCAAA |
| ***Zeastar*** | qRT-PCR | Endo1-F-ol 841 | 60 | 94 | CACAACGCCTTCAGCACCTA |
|  |  | Endo1-R-ol 842 |  |  | AAGGTGACGAAGCCGAAGC |
