# Supplementary material for: QTLs and candidate genes for desiccation and abscisic acid content in maize kernels
Source: BMC Plant Biol. 2010 Jan 4;10:2. doi: 10.1186/1471-2229-10-2 (PMC2826337; doi:10.1186/1471-2229-10-2)
Supplement: Additional file 3 — Identification of two ZEP genes in maize. The rice ZEP gene (Os04 g0448900; black) was used in BLAST analysis to identify five putative homologs from maize (The Institute for Genomic Research ID OGAGIC79TC, AZM5_13314, AZM5_13316, AZM5_24223, AZM_13312 and AZM5_13315; light brown). Maize ESTs were found for all this maize genomic contigs, except for AZM5_13315. Sequencing of amplified gDNA and cDNA with specific primers showed that ZEP is encoded by only two genes in maize (deduced CDS indicated in blue). This was confirmed using the maize HTGS database (maize ZEP1, [GenBank:AC194845.3]; maize ZEP2, [GenBank:AC206194.3]; brown) and in agreement with recent reports [39,44,45]. We used yrGATE at PlantGDB http://www.plantgdb.org/prj/yrGATE to annotate both genes(maize ZEP1: yrGATE_Zm2 gZEP1; maize ZEP2: yrGATE_Zm10 gZEP2; yrGATE_Zm10 gZEP2 gene annotation is incomplete since the last version of the maize HTGS database is not currently included in the software). [file 1471-2229-10-2-S3.DOC]

**Additional file 3 -** Identification of two *ZEP* genes in maize.


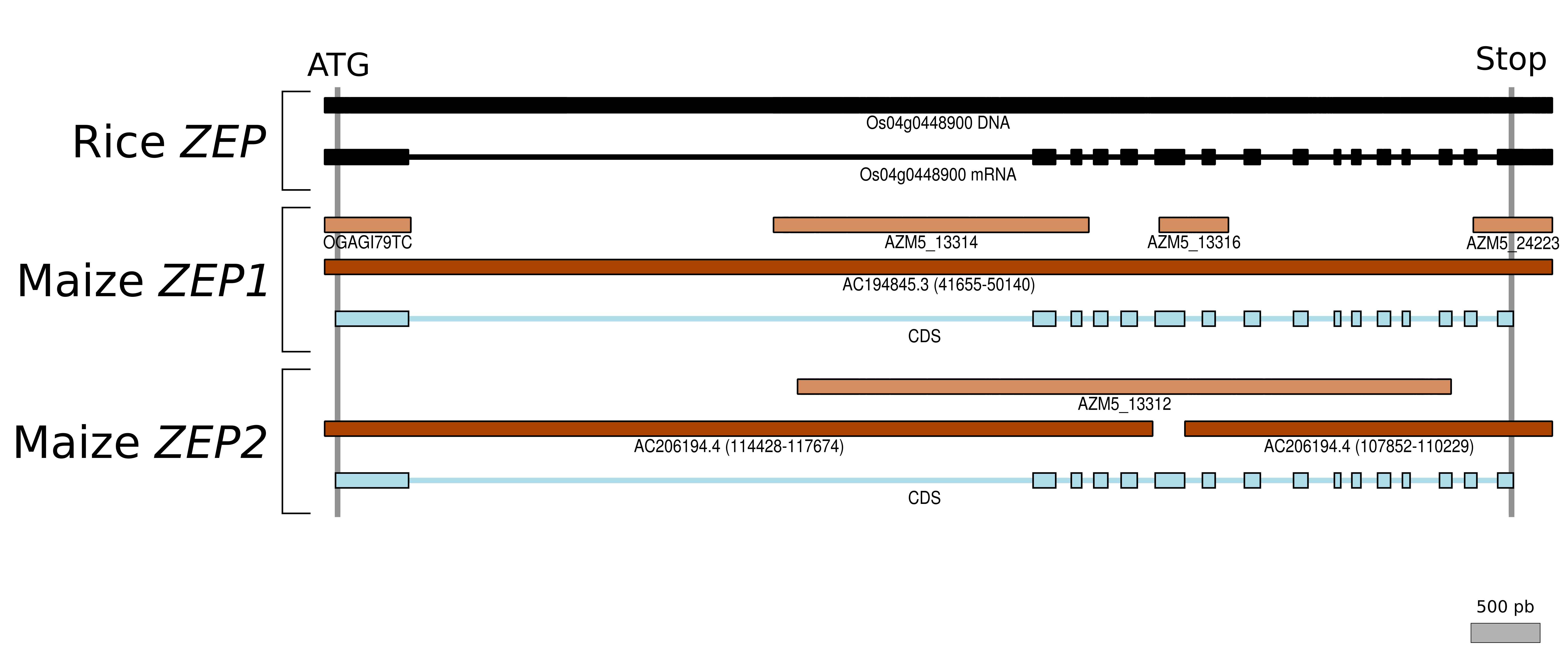
The rice *ZEP* gene (Os04g0448900; black) was used in BLAST analysis to identify five putative homologs from maize (The Institute for Genomic Research ID OGAGIC79TC, AZM5_13314, AZM5_13316, AZM5_24223, AZM_13312 and AZM5_13315; light brown). Maize ESTs were found for all this maize genomic contigs, except for AZM5_13315. Sequencing of amplified gDNA and cDNA with specific primers showed that ZEP is encoded by only two genes in maize (deduced CDS indicated in blue). This was confirmed using the maize HTGS database (maize *ZEP1*, [GenBank:AC194845.3]; maize *ZEP2*, [Genbank:AC206194.3]; brown) and in agreement with recent reports [39, 44, 45]. We used yrGATE at PlantGDB (<http://www.plantgdb.org/prj/yrGATE> ) to annotate both genes (maize *ZEP1*: yrGATE_Zm2gZEP1; maize *ZEP2*: yrGATE_Zm10gZEP2); Zm10gZEP2 gene annotation is incomplete since the last version of the maize HTGS database is not currently included in the software).
